# An engineered IL-2 reprogrammed for anti-tumor therapy using a semi-synthetic organism

**DOI:** 10.1038/s41467-021-24987-9

**Published:** 2021-08-09

**Authors:** Jerod L. Ptacin, Carolina E. Caffaro, Lina Ma, Kristine M. San Jose Gall, Hans R. Aerni, Nicole V. Acuff, Rob W. Herman, Yelena Pavlova, Michael J. Pena, David B. Chen, Lilia K. Koriazova, Laura K. Shawver, Ingrid B. Joseph, Marcos E. Milla

**Affiliations:** Synthorx, Inc., a Sanofi Company, La Jolla, CA USA

**Keywords:** Biotechnology, Synthetic biology, Drug discovery

## Abstract

The implementation of applied engineering principles to create synthetic biological systems promises to revolutionize medicine, but application of fundamentally redesigned organisms has thus far not impacted practical drug development. Here we utilize an engineered microbial organism with a six-letter semi-synthetic DNA code to generate a library of site-specific, click chemistry compatible amino acid substitutions in the human cytokine IL-2. Targeted covalent modification of IL-2 variants with PEG polymers and screening identifies compounds with distinct IL-2 receptor specificities and improved pharmacological properties. One variant, termed THOR-707, selectively engages the IL-2 receptor beta/gamma complex without engagement of the IL-2 receptor alpha. In mice, administration of THOR-707 results in large-scale activation and amplification of CD8^+^ T cells and NK cells, without Treg expansion characteristic of IL-2. In syngeneic B16-F10 tumor-bearing mice, THOR-707 enhances drug accumulation in the tumor tissue, stimulates tumor-infiltrating CD8^+^ T and NK cells, and leads to a dose-dependent reduction of tumor growth. These results support further characterization of the immune modulatory, anti-tumor properties of THOR-707 and represent a fundamental advance in the application of synthetic biology to medicine, leveraging engineered semi-synthetic organisms as cellular factories to facilitate discovery and production of differentiated classes of chemically modified biologics.

## Abstract (web summary)

The use of synthetic organisms could provide opportunities for discovery and advanced manufacturing of medical drugs. Here the authors use a semi-synthetic organism with an expanded genetic code to generate site-specific chemical modifications in human IL-2.

## Introduction

The recombinant human cytokine interleukin-2 (rhIL-2, or aldesleukin), originally approved for renal cell carcinoma in 1992, pioneered the era of immune oncology. Despite complete and durable responses, the widespread use of rhIL-2 therapy for oncology has been limited due to its severe toxicity^1–3^. The toxic side-effects of rhIL-2 therapy stem from two distinct and fundamental characteristics of the drug: (1) short half-life, requiring high doses for activity and (2) the engagement of the IL-2 receptor alpha subunit (IL-2Rα, or CD25) on off-target cells^4,5^. Therefore, engineering rhIL-2 to improve its half-life and decrease IL-2Rα engagement may produce a safer and more efficacious drug.

IL-2 functions as a lymphocyte growth and stimulatory factor, signaling via activation of the heterodimeric IL-2 receptor complex consisting of two subunits, IL-2 receptor β (CD122) and the common γ receptor subunit (CD132), or the heterotrimeric IL-2 receptor complex that includes IL-2 Rα (CD25). IL-2 activation of the IL-2 Rβγ complex induces proliferative and activation signals through STAT5 phosphorylation that drive effector T and natural killer (NK) cell expansion and tumor clearance^6^. In addition to the IL-2 Rβ and γ subunits, certain cell types express the IL-2 Rα subunit, which functions to increase affinity and capture of IL-2 by the signaling complex. The expression of the IL-2 Rα subunit on CD4^+^ regulatory T cells (Tregs) results in the preferential stimulation of these immunosuppressive cell types and is reported to limit the stimulatory effects of IL-2 on effector populations needed for anti-tumor responses^7,8^. In addition, IL-2 Rα is expressed on vascular endothelial tissue, type 2 innate lymphoid cells and eosinophils. Stimulation of these off-target cell types via aldesleukin treatment is thought to induce a constellation of severe adverse manifestations associated with vascular leak syndrome (VLS), an often fatal condition that results from increased capillary permeability^5,9^. Therefore, significant ongoing efforts aim to develop modified rhIL-2 variants that mediate potent IL-2 Rβγ stimulation of CD8 effector T and NK cells, yet reduce IL-2 Rα binding to mitigate Treg-mediated suppression and off-target toxicity^10^.

**Fig. 1:**
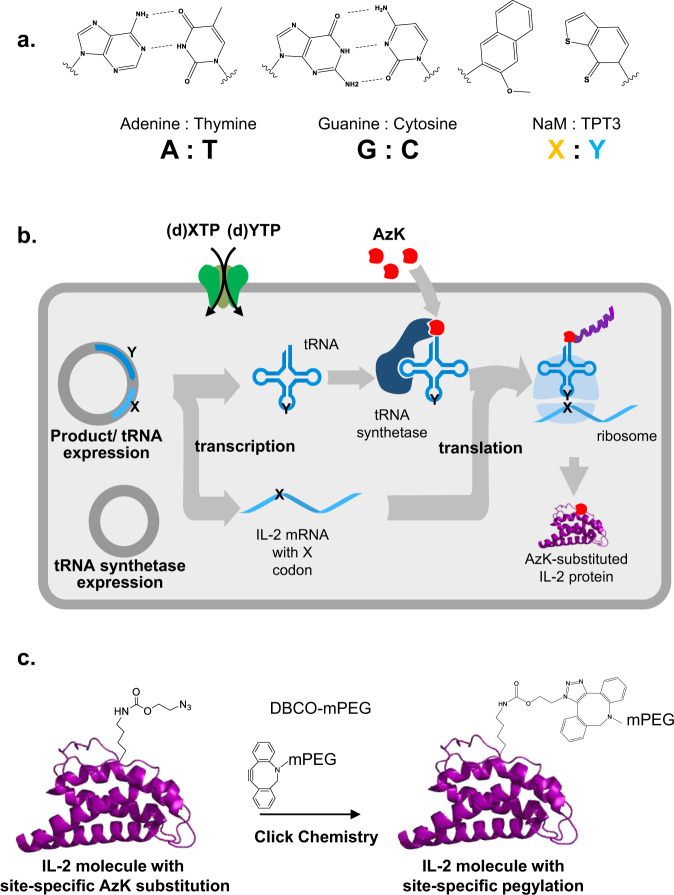
Overview of the X–Y expanded genetic code technology. **a** Structures of the natural base pairs A-T (left) and G-C (center) and the X–Y synthetic DNA base pair (right, highlighted in orange/blue). Rather than utilizing hydrogen bond donors and acceptors between pairs (dotted lines), the synthetic base pair formed between NaM (X) and TPT3 (Y) utilizes non-Watson–Crick mode of pairing that is based predominantly on hydrophobic packing interactions. **b** Schematic of the semi-synthetic organism (SSO) platform based on *E. coli* BL21(DE3). The organism constitutively expresses a variant of the PtNTT2 nucleotide transporter (green)^11^, which mediates the transport of X and Y nucleoside triphosphates from the growth medium into the cytoplasm for DNA and RNA synthesis. X–Y containing DNA sequences are transcribed into mRNA and tRNA that contain the codon (AXC) and anticodon sequences (GYU), respectively. The orthogonal tRNA synthetase PylRS from *M. barkeri* (blue) specifically recognizes the AzK (red shape) provided in the culture medium and charges this residue onto the corresponding GYU anticodon-containing orthogonal *pylT* tRNA. Finally, the cell’s ribosomal translation machinery utilizes the resulting pools of AzK-charged tRNA (GYU) to specifically decode the AXC codon, allowing the production of site-specifically AzK-modified protein product (purple chain with red shape) in concert with the native translational machinery. **c** Schematic showing an example AzK- IL-2 precursor molecule (purple chain with AzK incorporated). Reaction of the AzK-containing IL-2 proteins with DBCO-mPEG generates the site-specific and covalent attachment of the mPEG moiety to the AzK (one regioisomeric species shown). An array of different IL-2 variants with AzK incorporated into distinct sites was created and the scheme shown was employed to pegylate each variant.

We applied a synthetic biology platform^11^ to engineer an improved and differentiated version of rhIL-2. This technology allows the facile design and production of proteins modified with non-natural amino acids at specific sites, enabling targeted bioconjugation for modulation of pharmacology and drug properties. The technology relies on an *E. coli* BL21(DE3)-based strain engineered to harbor a synthetic, orthogonal DNA base pair formed between the synthetic nucleosides dNaM and dTPT3 (Fig. 1a), herein referred to as X and Y, respectively. The X–Y base pairs work in concert with the native A-T/G-C base pairs to expand the coding capacity of DNA, allowing the formation of unique and orthogonal codons in mRNA and the cognate anticodons in orthogonal tRNAs required to decode them. The new codons are matched with non-natural amino acids via an orthogonal tRNA synthetase (PylRS) from *M. barkeri*, which exclusively aminoacylates the *pylT* tRNA with the click-chemistry compatible, azide-containing non-natural amino acid (nAA) N6-(2-azidoethoxy)-carbonyl-l-lysine^12^ (hereafter referred to as AzK). In concert with the native translational machinery, the engineered cell utilizes the AzK-charged *pylT* tRNA (GYU) to decode the cognate AXC codon (Fig. 1b), allowing the production of modified proteins with site-specific incorporation of AzK.

Using this semi-synthetic microbial platform, we generated a library of site-specific rhIL-2 variants by substitution of single amino acid residues comprising the IL-2/IL-2Rα interface with AzK. Azide-modified variants were then covalently bioconjugated with linear polyethylene glycol (PEG) moieties using strain-promoted azide–alkyne click chemistry^13^ (SPAAC, Fig. 1c) and screened using recombinant cells in vitro to identify compounds that retain IL-2 Rβγ potency but demonstrate reduced IL-2 Rα engagement, a “not-alpha” pharmacological phenotype. This screening identified THOR-707 (also termed SAR-444245), a pegylated rhIL-2 variant that shows comparable potency relative to unmodified rhIL-2 at the IL-2 Rβγ, but dramatic and specific reduction of IL-2 Rα engagement. In primary human lymphocytes, THOR-707 potently stimulated pSTAT5 signaling in CD8^+^ T and NK cells, yet compared to rhIL-2 showed a significant reduction in potency for pSTAT5 induction in Treg cells.

In naive mice, THOR-707 demonstrated increased half-life and exposure relative to rhIL-2. THOR-707 administration stimulated the expansion of peripheral CD8^+^ T and NK cell counts without significantly expanding CD4^+^ Treg cells, even at doses expected to drive near-maximal receptor occupancy. In C57BL/6 mice bearing B16-F10 tumors, THOR-707 showed increased intratumoral distribution and retention and stimulated a significant elevation in infiltrating CD8^+^ T and NK cells and dose-dependent reduction in tumor growth. Together, these results suggest that site-specific bioconjugation of rhIL-2 with PEG to generate THOR-707 reprograms the pharmacological activity of this cytokine, modulating both half-life and receptor engagement profiles to create a potent CD8^+^ effector T and NK cell activator with reduced CD4^+^ Treg bias.

## Results

### Identification of pegylated IL-2 compounds that mediate potent IL-2 Rβγ agonism with reduced IL-2 Rα engagement

**Fig. 2:**
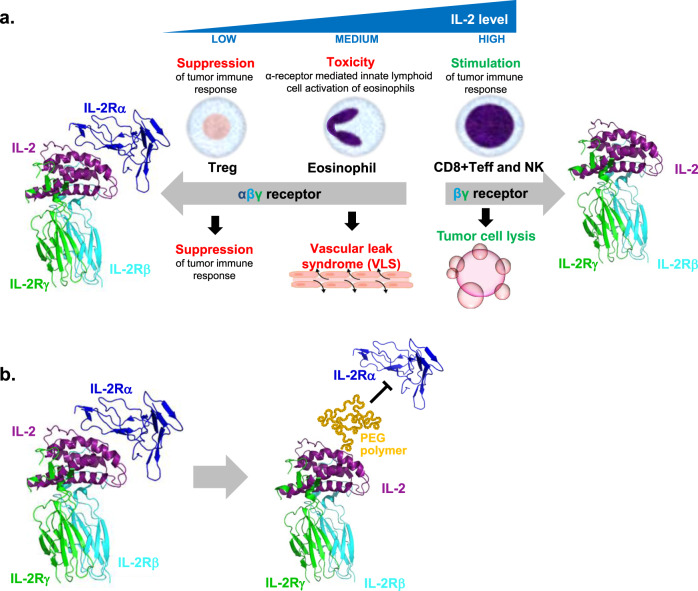
Overview of IL-2 pharmacology and therapeutic hypothesis of pegylated IL-2 with “not alpha” receptor specificity. **a** Schematic depicting IL-2 receptor biology, highlighting the desired and undesired effects of IL-2 therapy. To stimulate anti-tumor responses, high IL-2 (purple chain) concentrations are required to agonize the IL-2Rβγ complex (cyan and green chains) on CD8^+^ T and NK cells, driving activation and proliferation needed for tumor destruction. However, immune cell types with undesired biological activities also express the IL-2Rα chain, allowing their response to lower IL-2 concentrations. This results in Treg-mediated suppression of effector T cell populations, or stimulation of ILC-2 cells (not shown), which release IL-5 to drive VLS indirectly via eosinophil activation^9^. Therefore, the high rhIL-2 concentrations required to drive efficacy also result in Treg activation and toxicity in an IL-2Rα dependent manner. Blocking engagement of IL-2Rα may provide an improved therapy with reduced undesired target activation. **b** Concept of a site-specific PEG-modified “not alpha” IL-2, in which PEG attachment at the IL-2Rα interface blocks IL-2Rα engagement. The resulting compound allows normal IL-2Rβγ signaling and extends half-life via increased molecular size (not shown). Schematic of IL-2 (left) and THOR-707 (right) binding to the IL-2 receptor complex. Human IL-2 (purple chain) forms a heterotrimeric complex with the IL-2 receptor β (cyan), γ (green) and α (blue) subunits. Site-specific PEG modification (orange polymer) of THOR-707 at the IL-2 Rα receptor interface allows targeted reduction of the IL-2 Rα engagement without specific reduction in the engagement of the IL-2 Rβγ complex.

Structures of the IL-2/heterotrimeric receptor signaling complex^14,15^ demonstrated that the IL-2 Rα and βγ subunits engage IL-2 from opposite sides and that the α subunit engages IL-2 independently of the IL-2R βγ interfaces. We hypothesized that replacement of IL-2 residues in or near the IL-2 Rα interface with nAAs and pegylation of these sites could produce a modified IL-2 that reduces IL-2 Rα interactions while maintaining interactions with IL-2 Rβγ (Fig. 2a, b). Ten IL-2 residues hypothesized to affect IL-2 interactions with IL-2Rα after pegylation were selected for production and functional analysis: K35, R38, T41, F42, K43, Y45, E62, P65, E68 and V69. Compounds with the selected AzK-modified sites were expressed as inclusion bodies in our *E. coli-*based semi-synthetic organism, purified and refolded (Methods). The refolded AzK-modified IL-2 variants were then pegylated site-specifically at the AzK residue using DBCO-functionalized mPEG molecules, which allow covalent attachment of mPEG moieties to the AzK via copper-free click chemistry (Fig. 1c and “Methods” section).

Candidate compounds were screened for selective IL-2 receptor agonist activity using a recombinant human cell-based reporter system that expresses the IL-2 receptor β (IL-2Rβ) and γ (IL-2Rγ) subunits each fused to half of the split reporter enzyme β -galactosidase and a second cell line that additionally expresses the IL-2Rα subunit (Discoverx, Fremont CA, Supplementary Fig. 1A). Parallel testing with these cell lines allowed assessment of PEG-IL-2 variant activation of the IL-2 receptor βγ as well as the trimeric αβγ complex. The EC_50_ (potency) of each test article and comparison of dose–response curve profiles between IL-2 Rα positive and negative cell types allowed determination of the contribution of IL-2 Rα to the observed activity.

**Fig. 3:**
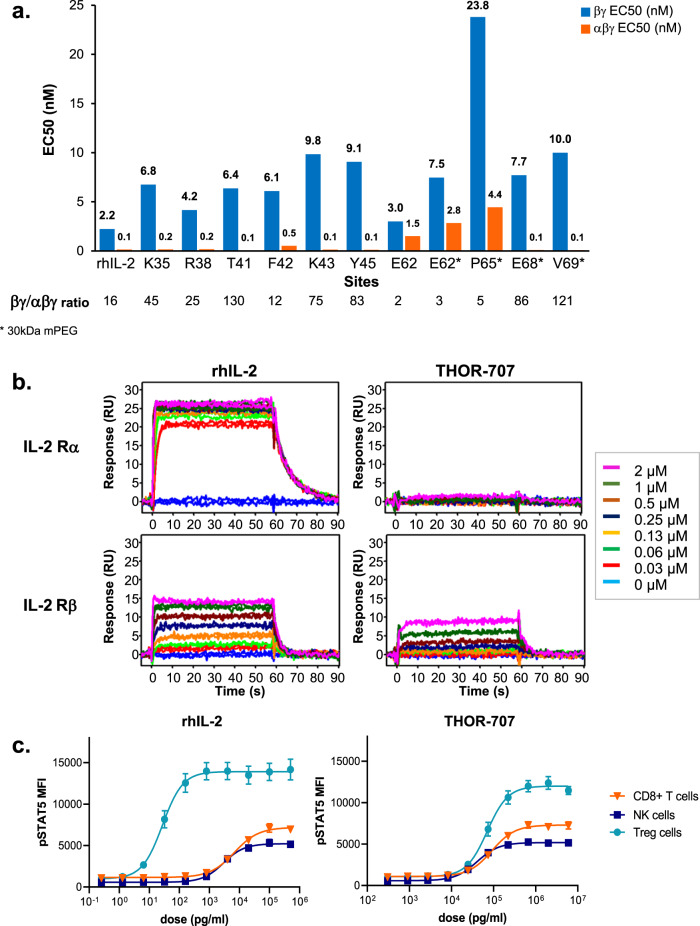
Identification and in vitro characterization of THOR-707, a pegylated IL-2 with “not alpha” pharmacology. **a** Cell-based screening of pegylated IL-2 variants identified compounds with a reduced contribution of IL-2Rα engagement to signaling potency. Pegylated variants were analyzed in parallel in the Discoverx PathHunter® IL-2 receptor agonist assays. Compounds were tested in duplicate in a dose–response format. Shown are the potency statistics (EC50) for each compound in the IL-2 receptor βγ (orange) and αβγ (blue) assays. Initially, pegylated variants were tested with a 10 kDa mPEG modification (compounds not marked with asterisk). During the screen, it was determined that larger mPEG modifications enable improvements in pharmacokinetics and target cell expansion (not shown), and larger mPEGs (30 kDa) were therefore employed for the remaining compounds (compounds marked with an asterisk). The calculated βγ: αβγ EC50 ratios for each test compound are listed below each plot. (*n* = 2 independent experiments). **b** Biochemical characterization of THOR-707 interactions with IL-2 Rα and β receptor subunits using SPR demonstrate “not alpha” signature of THOR-707. Human IL-2 Rα (top row) and β (bottom row) extracellular domains were immobilized on the surface of a SPR sensor and probed with a dilution series starting at 2 µM of either rhIL-2 (left column) or THOR-707 (right column). Test samples were injected for 60 s to allow measurement of association, followed by buffer only (wash, initiation time at 60 s) to measure dissociation kinetics. Response units (RU, *y* axis) are plotted versus time (s, *x* axis). Colors correspond to test concentration as shown (inset). **c** The cell-specific signaling of THOR-707 in human primary lymphocytes shows THOR-707 reprogramming to reduce IL-2Rα-mediated bias for Treg potency. Human PBMC samples from 6 healthy donors were stimulated in triplicate with concentration series of rhIL-2 (left panel) or THOR-707 (right panel). Treated cell populations were analyzed using multi-color flow cytometry to detect and quantify pSTAT5 activation in different T and NK cell subsets (cyan, blue, orange represent the data from Tregs, NK, and CD8^+^ T cells, respectively). The plots shown represent the average pSTAT5 signal, fit to a baseline restrained 4-parameter logistic regression and normalized to maximum signal to facilitate comparison of different cell subsets, with error bars representing SEM. (*n* = 6 independent donor samples). Source data are provided as a Source Data file.

From the screen, multiple pegylated IL-2 variants were identified that retained potent IL-2Rβγ agonist activity with reduced interaction with IL-2Rα: notably, variants replacing positions Glu62 and Pro65 (Fig. 3a and Supplementary Table 1, Supplementary Fig. 1). Initial studies demonstrated similar receptor specificity, but increased half-life and pharmacodynamic responses with 30 kDa mPEG modification in mice (not shown) and therefore the 30 kDa mPEG was selected for further screening and development. The IL-2 compound with residue Pro65 replaced with AzK and pegylated with a 30 kDa mPEG, hereafter referred to as THOR-707, exhibited higher expression levels of the precursor protein and was selected for further characterization. In the IL-2Rβγ assay, the EC50 of the THOR-707 compound was moderately reduced in potency compared to rhIL-2 (~4-fold, 120 vs 32 ng/mL, respectively, Supplementary Fig. 1). However, in the IL-2Rαβγ assay, the potency of THOR-707 was strongly reduced relative to rhIL-2 (~47-fold, 33 vs 0.7 ng/mL, respectively, Supplementary Fig. 1).

### Biochemical characterization of THOR-707 interactions with IL-2 receptor α and β subunits using SPR

The biochemical interactions of THOR-707 with human IL-2 receptor subunits were measured using surface plasmon resonance (SPR). The extracellular portions of the human IL-2 receptor subunits α and β were immobilized on the surface of SPR sensor chips. Concentration series of rhIL-2 or THOR-707 were applied to these surfaces and receptor interactions were monitored as the change in response units (RU) as a function of time during association and dissociation phases. On IL-2Rα sensors, rhIL-2 showed a rapid association and slow dissociation kinetics, demonstrating high-affinity binding (Fig. 3b, upper left panel, Supplementary Table 2). In contrast, THOR-707 showed negligible interactions with the IL-2Rα surface even at the highest test concentration of 2 µM (Fig. 3b, upper right panel, Supplementary Table 2). Surfaces containing immobilized IL-2 Rβ showed similar association and dissociation responses with both rhIL-2 and THOR-707, with estimated dissociation constants (K_D_) values of 0.285 and 1.96 µM, respectively (Supplementary Table 2). A THOR-707 compound containing a smaller (5 kDa) mPEG showed similarly negligible IL-2 Rα engagement, but 6-fold higher affinity to the IL-2 Rβ subunit (Supplementary Fig. 2 and Supplementary Table 2), suggesting the modest difference in *K*_D_ for the β subunit observed between compounds is likely due to non-specific shielding effects of the large PEG as observed for other pegylated proteins^16^. These results suggest that THOR-707 is specifically defective in IL-2Rα interactions while largely retaining binding to IL-2Rβ.

### Reprogrammed cell-specificity of THOR-707 for pSTAT5 signaling in primary human lymphocytes

To determine how the differential receptor specificity of THOR-707 affects activation of primary immune cell subpopulations, we profiled lymphocyte activation in human peripheral blood mononuclear cell (PBMC) samples using multi-color flow cytometry. Fresh Leukocyte Reduction System (LRS)-derived PBMC samples were treated with either rhIL-2 or THOR-707 in 5-fold dilution series starting with a top concentration of 30 µg/mL. After a 45 min incubation, samples were fixed and stained with antibodies to detect the phosphorylated form of the transcription factor STAT5 (pSTAT5), a marker of upstream engagement and activation of IL-2 receptor signaling complexes and a panel of surface markers to follow pSTAT5 formation in specific T and NK cell subpopulations.

In NK and effector T cell (CD3^+^ CD8^+^) populations, THOR-707 retained potency relative to rhIL-2, with EC_50_ values for pSTAT5 induction within 5–10-fold of the native cytokine (Fig. 3c and Supplementary Table 2). In contrast, the EC_50_ value for THOR-707-induced pSTAT5 signaling in the Treg subpopulation (CD3^+^ CD4^+^ IL-2Rα^+^ CD127^−^) was reduced by >2000-fold compared to rhIL-2 (Fig. 3c and Supplementary Table 3). This substantial decrease in potency for THOR-707 specifically in the Treg population indicates that pegylation of IL-2 at position 65 allows potent agonism of IL-2 receptors yet eliminates the strong bias of IL-2 for Treg stimulation relative to effector T cells. Indeed, the Teff/Treg EC_50_ ratio for rhIL-2 was 216 (7.5 vs 0.035 ng/mL) compared to 1.3 (92.5 vs 73 ng/mL) for THOR-707. These results demonstrate that THOR-707 is a potent IL-2Rβγ agonist with a greatly reduced bias for stimulating IL-2Rα-expressing Treg cells.

### Reduction in THOR-707 engagement of IL-2 Rα is mediated by AzK substitution at P65 and pegylation

The proline residue at position 65 in human IL-2 appears to impart a significant bend in helix H3, a region that mediates substantial interactions with IL-2 Rα^14,15^. As the sequence of THOR-707 contains a substitution of this proline to a lysine-like residue, we reasoned that this modification might result in a distinct helical conformation that mis-aligns residues critical for IL-2 Rα engagement. To elucidate the tertiary structure of the THOR-707 protein component, the crystal structure of the human IL-2 variant P65K was solved at 1.8 Å resolution (Supplementary Fig. 3B and Supplementary Tables 4, 5). The lysine residue incorporated at position 65 was selected to mimic the effect of the AzK substitution of this position on protein structure, as lysine constitutes the majority of the backbone of the AzK residue without the excessive hydrophobicity of the un-conjugated azide (Supplementary Fig. 3A). Surprisingly, the structure of the IL-2 P65K was remarkably similar to that of rhIL-2. Structural alignments show that the lysine substitution for proline at position 65 did not result in large-scale changes to the IL-2 tertiary structure, including the conformation of helix H3 (Supplementary Figs. 3B and 4A). Consistent with the above biochemical and ex vivo data, an inspection of the P65K region in the structure shows that this modification occupies a region of the IL-2/IL-2 Rα interface that mediates productive interactions (Supplementary Fig. 4B, C). Together, these results suggest that steric effects of the AzK and potentially the PEG modifications largely drive the reduction in IL-2 Rα potency of THOR-707.

To determine the relative contributions of proline 65 substitution with AzK and pegylation of this residue to the reduction in IL-2 Rα engagement and IL-2 R potency, we generated variants with structural elements of the THOR-707 modifications, including replacement of proline 65 with AzK (without PEG attachment), pegylation with a 5 kDa mPEG and the full THOR-707 compound with 30 kDa mPEG modification. The cell-specific potency for each compound was measured using the human PBMC assay for pSTAT5 activation using flow cytometry described above. At CD8^+^ T cells, the potency for pSTAT5 induction by rhIL-2 was 1.2-fold higher than P65 AzK (14,048 vs 16,908 pg/mL respectively, Supplementary Fig. 5), confirming that substitution of P65 with AzK residue has negligible effects on potency at the IL-2 Rβγ. The pegylated compounds with 5 kDa and 30 kDa mPEG substituents demonstrated an additional 3.5 to 7.4-fold decrease in potency at CD8^+^ T cells (48,904 vs 125,442 pg/mL, respectively, Supplementary Fig. 5), suggesting PEG has general and non-specific effects on potency.

In contrast, at Treg cells, which express the IL-2 Rα subunit in addition to the IL-2 Rβγ, the potency for pSTAT5 induction by rhIL-2 was ~175-fold higher than P65 AzK (31.1 vs 5446 pg/mL, respectively, Supplementary Fig. 5) demonstrating that AzK modification itself can contribute substantially to reduction in IL-2Rα engagement. Furthermore, the pegylated THOR-707 compounds showed a modest reduction in potency at Treg cells similar in magnitude to the potency reductions at CD8^+^ T cells, (5 vs 11.5-fold, respectively for 5 and 30 kDa mPEG variants of P65, Supplementary Fig. 5). The similar stepwise and minor reductions in potency of PEG size variants in both CD8^+^ T cells and Tregs suggest that PEG polymers are not specifically required to prevent IL-2 Rα engagement, but may impact potency in THOR-707 via non-specific envelopment as observed with other pegylated cytokines^16^. Together, these results suggest that steric effects mediated largely by the substitution of proline 65 with AzK result in the reduction in IL-2 Rα engagement by THOR-707.

### THOR-707 demonstrates increased half-life and improved plasma pharmacokinetics in mice

Due to the large increase in molecular weight, the attachment of a large 30 kDa mPEG polymer to THOR-707 was expected to affect the exposure of the molecule in vivo. To evaluate the pharmacokinetic properties and distribution of THOR-707 compared to rhIL-2, single intravenous doses were administered to naive C57BL/6 mice. Following administration, the THOR-707 exposure was 200 times higher than aldesleukin (AUC_0–*t*_, Supplementary Fig. 6, Supplementary Table 6). In addition, THOR-707 demonstrated a 23-fold extended terminal *t*_1/2_ compared to aldesleukin (13.3 vs. 0.57 h, respectively, Supplementary Table 6). The THOR-707 volume of distribution at steady state (Vss; 82.4 mL/kg) was about 4.7-fold reduced relative to aldesleukin and similar to the blood volume for mice^17^ (85 mL/kg), suggesting that THOR-707 is mostly distributed within the systemic circulation as is typical of pegylated proteins^18^.

### THOR-707 drives sustained pSTAT5 signaling, Ki-67 expression and expansion of CD8^+^ T and NK cells in naive mice

**Fig. 4:**
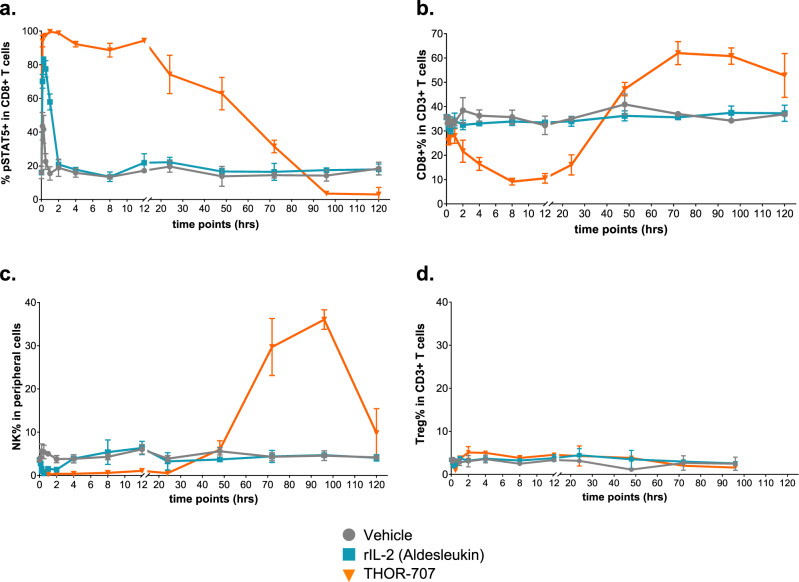
A single dose of THOR-707 drives sustained pSTAT5 signaling, extravasation and proliferation of CD8^+^ T and NK cells without significant Treg expansion in naive mice. A single intravenous bolus dose of 0.3 mg/kg rhIL-2 or THOR-707 was administrated to naive mice and blood was drawn via cardiac puncture at the indicated time points. Immune cell identification and signaling were assessed by flow cytometry, each data point represents an average of 3–4 replicates at each time point ± standard deviation (SD). **a** Percentage peripheral blood CD8^+^ T cells that are pSTAT5 positive. **b** Percentage of peripheral CD3^+^ cells that are CD8 positive. **c** Percentage of singlets (peripheral blood cells sampled) that are NK cells. **d** Percentage of CD3^+^ cells that are CD4^+^ Treg cells (*n* = 4 animals). Source data are provided as a Source Data file.

The increased exposure observed for THOR-707 relative to rhIL-2 in mice suggested that THOR-707 may drive sustained signaling and result in an increased expansion of target CD8^+^ T and NK cells. To measure and compare the pharmacodynamics of these compounds, peripheral blood samples collected from naive mice dosed with THOR-707 or rhIL-2 were analyzed using multi-parametric flow cytometry measuring cell-specific signaling and proliferation. THOR-707 dosing induced persistent pSTAT5 signaling in peripheral blood cell populations up to ~72 h for CD8^+^ T cells and 96 h for NK cells, while pSTAT5 in these cell types returned to baseline after only 2 h in mice dosed with a molar equivalent of aldesleukin (Fig. 4 and Supplementary Fig. 7). Due to the high-affinity binding of aldesleukin to IL-2Rα, pSTAT5 induction in Treg cells lasted for about 24 h before returning to the baseline (Supplementary Fig. 8). In contrast, the increased exposure of THOR-707 extended pSTAT5 signaling in Tregs, but was similar in duration to that of CD8^+^ T and NK cells, consistent with normal IL-2 Rβγ signaling without IL-2 Rα binding.

In addition to pSTAT5 induction, a single dose of THOR-707 induced the rapid disappearance of CD8^+^ T and NK cells from the peripheral blood within the first 10 h post-administration, suggesting activation and extravasation of these cells as previously demonstrated for rhIL-2 (Foreau et al.^19^). Beginning 12–24 h post-dose, THOR-707 induced expression of Ki-67, a nuclear protein marker for cell proliferation, in all three cell populations (CD8^+^ T, NK and Treg cells) to a similar degree across all dose levels, while induction of Ki-67 with aldesleukin treatment was not observed (Supplementary Fig. 9). THOR-707 resulted in proliferative responses of NK and CD8^+^ T cells (Fig. 4b, c, respectively) and phenotypic analysis of the resulting CD8^+^ T cell population using flow cytometry revealed substantial expansion of CD44^+^ memory cells within this population (Supplementary Fig. 10). The proliferative response in CD8^+^ T and NK cells was not observed in the rhIL-2 treatment group. Finally, THOR-707 dosing stimulated only low and transient levels of Treg cell expansion (Fig. 4d). These results suggest that the increased exposure of THOR-707 drives extended pSTAT5 signaling, extravasation and proliferation of peripheral CD8^+^ T and NK cells, without driving a significant expansion of peripheral Treg cells in naive mice.

### THOR-707 elicits CD8^+^ T and NK cell proliferation with reduced IL-5 release, a biomarker for VLS, relative to high-dose IL-2

The pronounced pharmacodynamic response induced by THOR-707 administration to mice demonstrates that this compound is an efficient inducer of CD8^+^ T and NK cell proliferation. Previous studies demonstrated that CD25 engagement by IL-2 is required to induce vascular leak syndrome^5,9^, a severe adverse event observed with high-dose IL-2 therapy. To study whether THOR-707, a “not alpha” IL-2 may have the potential for an improved safety profile, naive mice were administered THOR-707 or aldesleukin and plasma samples were collected at intervals post-dosing to monitor CD8^+^ T and NK cell proliferation via flow cytometry, as well as plasma IL-5 cytokine levels, a biomarker for severe rhIL-2 toxicity, specifically VLS^9^. Under these conditions, a single dose of THOR-707 induced significant CD8^+^ T and NK cell proliferation that exceeded levels induced by rhIL-2 (Aldesleukin, Supplementary Fig. 11A, B). However, plasma IL-5 levels in response to THOR-707 were substantially lower compared to those induced by rhIL-2 (Supplementary Fig. 11C, D and Supplementary Table 7). These results confirm that, compared to rhIL-2, THOR-707 drives stronger activation and expansion of peripheral effector T and NK cell populations while inducing significantly lower levels of IL-5, which promotes VLS^9^.

### THOR-707 shows enhanced accumulation and retention in tumor tissue relative to plasma and other tissues

The pharmacokinetic properties and distribution of THOR-707 in tumor tissue versus plasma were analyzed in C57BL/6 mice bearing subcutaneous B16-F10 tumors. Following a single intravenous dose, THOR-707 exposure in both the plasma as well as the tumor increased in an approximately dose-proportional manner (Supplementary Table 8, Supplementary Fig. 12). The *t*_1/2_ in tumor tissue was nearly twice that in plasma (24.6 vs. 12.6 h for the 3 mg/kg dose group), indicating that THOR-707 distributes into the tumor and is retained intratumorally for a longer duration relative to the blood compartment (Supplementary Fig. 12). To assess the specificity of THOR-707 accumulation in tumor relative to other tissues, THOR-707 levels were also analyzed in the splenic tissue from the THOR-707 dosed animals. After dosing, spleen exposure levels increased in an approximately dose-proportional manner and the apparent *t*_1/2_ of THOR-707 was shorter in the spleen compared to plasma or tumor (10.8 and 10.2 h in the 1 and 3 mg/kg dose groups, respectively, Supplementary Fig. 12 and Supplementary Table 8). These data demonstrate that THOR-707 distribution and retention in tumors is enhanced relative to organ tissue, consistent with observations with other pegylated proteins^20^.

### THOR-707 induces memory CD8 T cell proliferation and lymphocyte expansion in mouse B16-F10 tumors and controls tumor growth

**Fig. 5:**
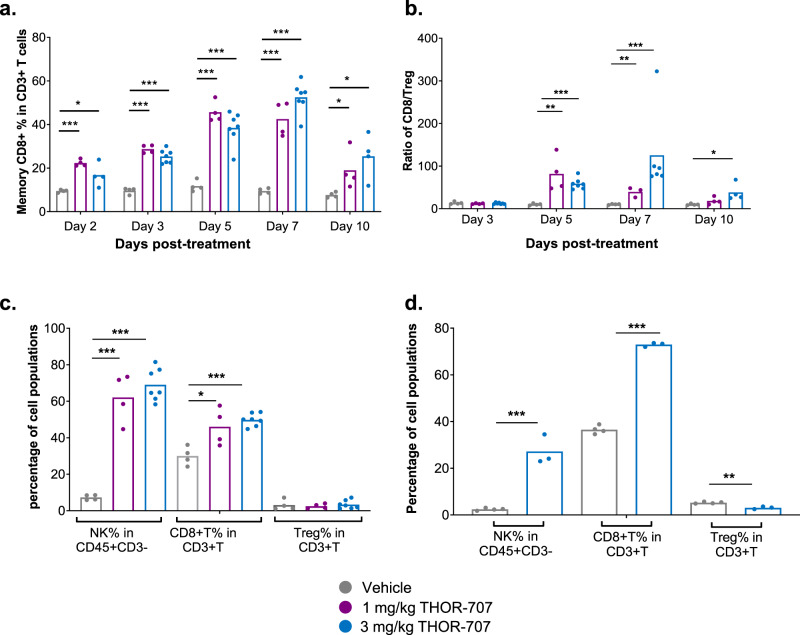
THOR-707 induced the expansion of peripheral, intratumoral and splenic CD8^+^ T and NK cells in B16-F10 tumor-bearing C57BL/6 mice. **a** Percentage of CD8^+^ memory T cells within the CD3^+^ T cell population up to 10 days post-dose of a single 1 or 3 mg/kg intravenous bolus dose of THOR-707 to tumor-bearing mice. Bars represent an average from 4 replicates at each time point (except *n* = 7 for 3 mg/kg at days 3, 5, 7), shown as individual points. *p < 0.05, ***p < 0.001 using unpaired, non-parametric *t*-test. **b** Ratio of CD8^+^ T cell vs. CD4^+^ Treg cells in the peripheral blood following administration of a single 1 or 3 mg/kg intravenous bolus dose of THOR-707 to B16-F10 tumor-bearing mice. Bars represent an average from 4 replicates at each time point (except *n* = 7 for 3 mg/kg at days 3, 5, 7), shown as individual points. *p < 0.05, **p < 0.01, ***p < 0.001 using unpaired, non-parametric *t*-test. **c** Percentage of NK cells in CD3^−^ T, CD8^+^ T and Treg cells in the tumor CD3^+^ T cell population following treatment with a single 1 or 3 mg/kg intravenous bolus dose of THOR-707. Tumor samples were analyzed for immune cell populations 5 days after treatment by FACS (data represented for day 5). Bars represent an average from 4 replicates at each time point (except *n* = 7 for 3 mg/kg at days 3, 5, 7), shown as individual points. *p < 0.05, **p < 0.01, ***p < 0.001 using unpaired, non-parametric *t*-test. **d** Percentage of NK cells in CD3^−^ cells, CD8^+^ T and Treg cells in the spleen CD3^+^ T cell population following treatment with a single 3 mg/kg intravenous bolus dose of THOR-707. Spleen samples were analyzed for immune cell populations 5 days after treatment by FACS. Bars represent an average of 4 replicates at each time point shown. *p < 0.05, **p < 0.01, ***p < 0.001 using unpaired non-parametric *t*-test. Source data are provided as a Source Data file.

The preferential accumulation of THOR-707 in tumor tissue suggested that this compound may drive additional pharmacodynamics within tumors. Peripheral blood, tumor tissue and splenic tissue was collected from B16-F10 tumor-bearing mice dosed with THOR-707 or aldesleukin and lymphocyte samples were collected and analyzed using multi-parametric flow cytometry to characterize cell-specific signaling and expansion. As in naive mice, B16-F10 tumor-bearing mice dosed with THOR-707 showed persistent pSTAT5 signaling in peripheral blood populations of CD8^+^ T and NK cells (Supplementary Fig. 7). Peripheral memory CD8^+^ T cell populations increased 4- to 5-fold in THOR-707-treated animals compared to vehicle (Fig. 5a) and conventional CD8^+^ T cells, NK cells and Treg populations showed responses comparable to those observed in naive animals dosed with THOR-707 (Supplementary Figs. 14 and 15). The robust CD8^+^ T cell proliferation without Treg expansion resulted in significant remodeling of the T cell population in the peripheral blood of these animals, as demonstrated by increased CD8/Treg ratios, peaking at ~85 at day 5 and ~120 at day 7 in the 1 and 3 mg/kg THOR-707 dose groups, respectively (Fig. 5b).

To assess the effect of THOR-707 dosing on tumor-infiltrating immune populations, B16-F10 tumor tissue and spleen tissue were collected at day 5 post-dosing and tissue-resident lymphocyte populations were isolated and characterized using flow cytometry. Analysis of tumor samples revealed significant expansion of both CD8^+^ T and NK cells within the tumors of THOR-707-treated mice relative to vehicle-treated mice (Fig. 5c). In contrast, the Treg cell population did not expand within the tumor relative to the vehicle control (Fig. 5c).

To determine whether THOR-707 also drives effector cell proliferation in, or trafficking to, lymphoid tissues, the effect of THOR-707 dosing on lymphoid cells in the spleen was characterized. Spleens were harvested on days 5 and 7 from B16-F10 tumor-bearing mice following a single 3 mg/kg dose of THOR-707. Similar to the tumor compartment, in the spleen, a marked expansion of CD8^+^ T and NK cells was observed in THOR-707-treated animals, while no significant change in CD4 Treg population was observed (Fig. 5d), resulting in a progressive increase in the splenic CD8^+^ T/Treg ratio at days 5 and 7 post-dose (Supplementary Fig. 13). Together, these results suggest that THOR-707 drives the proliferation of NK and CD8^+^ T cells in peripheral blood, tumor and spleen tissue without induction of suppressive CD4^+^ Treg subpopulations.

**Fig. 6:**
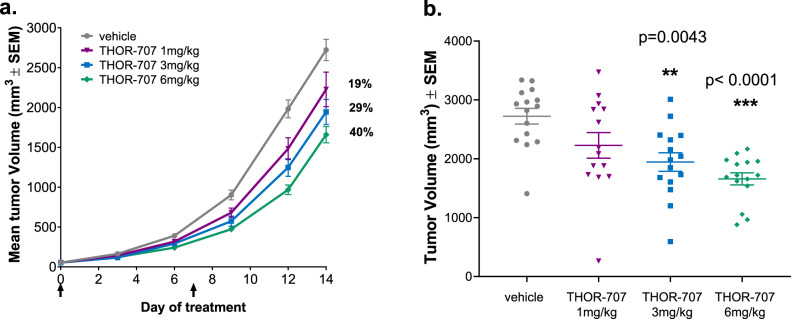
THOR-707 reduces B16-F10 tumor proliferation in C57BL/6 mice. **a** Mice implanted with B16-F10 tumors were administered vehicle or THOR-707 doses at 1, 3, and 6 mg/kg upon tumor size measuring 50 mm^3^ (day 0) and 1 week later (day 7). Mean tumor size (±SEM) in mm^3^ is plotted at 3-days intervals post-dose initiation of treatment, indicated by black arrows, and percentages indicate the percent reduction in mean tumor volume observed at day 14. **b** Tumor volumes (mm^3^) measured at day 14 post-dose initiation are plotted for different dose groups. Each point indicates an individual tumor, mean tumor volume (horizontal line with error bars representing ± SEM) is shown. *n* = 15 animals. **p = 0.0043, ***p < 0.0001 using one-way ANOVA. Source data are provided as a Source Data file.

In addition to inducing a significant expansion of CD8^+^ T cells in tumors, THOR-707 administration resulted in a dose-dependent inhibition of B16-F10 tumor growth (Fig. 6a). The tumor volumes from each dose group showed reduced kinetics of outgrowth with increasing dose, and scatter plots of individual tumor volumes at day 14 showed statistically significant anti-tumor activity, with mean tumor volume reductions of 29% (*p* = 0.043) and 40% (*p* < 0.001), compared to vehicle control at 3 and 6 mg/kg dose groups, respectively (calculated using one-way ANOVA, Fig. 6b). These results demonstrate that THOR-707 treatment as a monotherapy demonstrates the capacity to reduce B16-F10 tumor progression in mice.

## Discussion

To overcome the limitations of aldesleukin therapy, including expansion of Treg cells and induction of vascular leak syndrome, we applied a synthetic biology platform to engineer a potentially safer and more efficacious version of rhIL-2. Using an engineered organism with a semi-synthetic, six-letter DNA code, we generated a library of genetically encoded rhIL-2 variants with a site-specific azide-containing residue, then specifically and covalently pegylated these variants using click chemistry. This approach enabled the identification of pegylated IL-2 variants that were difficult or impossible to produce using standard methodologies such as site-directed muteins or lysine-mediated chemical coupling. Another method to incorporate non-natural amino acids into proteins includes stop codon suppression, which reappropriates a genetically redundant stop codon (typically amber UAG) to direct the site-specific incorporation of a non-natural amino acid into a polypeptide via an orthogonal tRNA/tRNA synthetase system. This method involves competition between release factor binding and tRNA-mediated decoding and results in abundantly expressed product truncations or requires extensive genome recoding and strain optimization to mitigate^21^. In contrast, the semi-synthetic organism technology presented here allows site-specific incorporation of a non-natural amino acid without competition with dedicated translation factors and may enable more streamlined processing due to higher yields and reduced truncated product formation. This technology may therefore represent a general tool for the engineering of enhanced biologics that are compatible with microbial production.

Applying the semi-synthetic organism technology to drug discovery, we generated and screened an array of peg-modified variants of IL-2 and discovered THOR-707, a pegylated IL-2 with differentiated pharmacological properties. Our in vitro and ex vivo studies demonstrated that THOR-707 is a potent stimulator of CD8^+^ T and NK cells, which express the IL-2 Rβγ complex. Compared to aldesleukin, THOR-707 showed a specifically reduced potency for stimulation of suppressive Treg cells, demonstrating that the pharmacology of THOR-707 is reprogrammed toward stimulation of effector populations required for a potent anti-tumor response. In mice, THOR-707 demonstrated an extended half-life and reduced volume of distribution compared to rhIL-2 (*V*_ss_ (mL/kg) Supplementary Table 6), suggesting that increased size and/or hydrodynamic radius of the pegylated compound reduces renal clearance rates. In tumor-bearing animals, THOR-707 was found to accumulate substantially and specifically within the tumor tissue (Supplementary Fig. 12 and Supplementary Table 8), increasing the exposure of the compound to the tumor microenvironment compared to other tissues such as the spleen. This tumor accumulation effect may be attributed to increased permeability of the inflamed vascular tissue within the tumor and we speculate that tumor retention may increase the local efficacy of the compound for effector cell expansion.

In addition to improved exposure and tissue distribution, THOR-707 administration elicited robust, cell-specific activation and proliferation of effector lymphocyte populations in mice. A single dose of THOR-707 increased memory CD8^+^ T and NK cell proliferation in the peripheral blood (Figs. 4 and 5). Despite pSTAT5 and Ki-67 induction in Tregs, THOR-707 administration did not induce Treg expansion (Figs. 4 and 5), suggesting that IL-2Rα-mediated signaling and/or additional stimulatory signals are required for either Treg proliferation or survival.

Previous studies demonstrated a requirement for IL-2Rα engagement for high-dose IL-2 induction of VLS, a severe and often fatal adverse event observed with IL-2 therapy^5,9^. As THOR-707 is a “not alpha” IL-2, we compared the capacity of THOR-707 and rhIL-2 for induction of plasma IL-5, a key mediator of eosinophil activation, shown by Bluestone and co-workers to be a strong biomarker for VLS^9^. Under conditions that drive improved CD8^+^ T and NK cell proliferation, THOR-707 induced significantly lower plasma IL-5 levels relative to rhIL-2 (Supplementary Fig. 11A–D and Supplementary Table 7). These results suggest that THOR-707 drives enhanced pharmacodynamic response in CD8^+^ T and NK cells with reduced potential for severe adverse events such as VLS compared to rhIL-2.

In B16-F10 tumor-bearing animals, THOR-707 administration drove the marked expansion of CD8^+^ T and NK cells within tumor and spleen tissues without stimulation of Treg expansion (Fig. 5 and Supplementary Figs. 9, 14 and 15). Tumor-bearing mice administered THOR-707 showed a dose-dependent and statistically significant reduction in tumor growth rate as measured by mean tumor size after only two doses of the compound (Fig. 6). Additional efficacy studies employing alternative tumor models may provide further insight into the anti-tumor activities of THOR-707 alone and in combination with immune-modulating compounds such as checkpoint inhibitors.

In addition to THOR-707, a variety of IL-2-based drugs that utilize alternative strategies for half-life extension and “not-alpha” pharmacology have been reported, several of which are currently under clinical investigation^10^. Approaches to reduce IL-2Rα engagement include IL-2 muteins containing amino acid substitutions to block IL-2Rα binding, non-specific and hydrolysable pegylation, IL-2Rα fusions and antibody complexes or fusions^10^. While clinical proof-of-concept for additional “not-alpha” IL-2 molecules in humans is growing, fusion molecules and muteins by definition create neo-epitopes that may generate an anti-drug immune response and potentially even antibodies against a patient’s endogenous protein^22^. In contrast, THOR-707 has been engineered to reduce the potential for immunogenic response: the PEG attachment is positioned within a region of the IL-2 molecule that has a weak propensity to provide the P1 anchors required for class II MHC presentation. The large mPEG attached to this position is covalent, highly stable and irreversible and expected to prevent processing at multiple steps in the MHC-II presentation pathway. Therefore, unlike alternative IL-2 compounds in development, the chemically modified region of THOR-707 may block immune surveillance pathways that could lead to anti-drug antibody formation.

Overall, this work represents a seminal step in synthetic biology and medicine- the application of a semi-synthetic genetic system for the engineering, discovery and manufacturing of an improved, chemically modified protein pharmaceutical candidate. Looking ahead, the myriad new codons afforded by the X–Y genetic code^23^ will likely enable multiple distinct chemical functionalities to be designed into proteins for increased, combinatorial complexity to overcome multiple distinct limitations of native protein drugs. What was historically the exclusive domain of small molecule and peptide synthesis, the capacity to apply medicinal chemistry-like approaches to engineer full-length recombinant proteins with unique functions will enable a new frontier in biological medicines.

## Methods

### Ethical approval statement

The studies performed by the authors reported herein that make use of human blood samples were collected from anonymous healthy donors and were approved by local institutional review boards. All subjects provided informed consent. The studies were performed following the guidelines of the World Medical Association’s Declaration of Helsinki. All procedures performed in studies involving animals reported here were conducted following guidelines for animal welfare which comply with the U.S. Department of Agriculture’s Animal Welfare Act (9 CFR Parts 1, 2 and 3) as applicable.

### Molecular biology reagents and assay materials

All primers for PCR amplification were purchased from Integrated DNA Technologies (Coralville IA). All unnatural oligonucleotides were synthesized by Biosearch Technologies (Petaluma, California, USA) with purification by reverse phase cartridge. Phosphoramidites of dNaM and dTPT3 were synthesized by WuXi AppTec (Tianjin China). The dNaM, dTPT3, NaM and TPT3 nucleosides were synthesized by WuXi AppTec and then triphosphorylated by MyChem LLC (San Diego CA). Analytical data for custom nucleotides are summarized in Supplementary Table 9 below. Plasmid clean-up for cloning plasmids was performed using QIAprep commercial kits (QIAGEN). All cloning enzymes were purchased from New England Biolabs (Ipswitch, MA). gBlock® gene fragments and natural oligonucleotides (with standard purification and desalting) were purchased from IDT (Coralville, IA). Sequencing was performed by Genewiz (San Diego, California, USA). Plasmids were isolated using commercial miniprep kits (QIAprep, Qiagen or ZR Plasmid Miniprep Classic, Zymo Research). PCR reactions for amplification of DNA used TempAssure PCR 8-Tube Strips (USA Scientific, Cat. No. 1402-2380) with a Roche Life Science LightCycler 96 Q-PCR thermocycler. DNA Clean and Concentrator kits from Zymo Research were used for purification of all Golden Gate Assembly reactions and Golden Gate vector and insert fragments. Both Golden Gate Assembly and Gibson Assembly protocols were performed on a MJ Research PTC-200 Thermal Cycler. DNA quantitation was carried out using Qubit DNA HS Assay Kits (Molecular Probes, Cat. No. Q32854). A list of primers used in these studies is included in Supplementary Table 10 below.

### Growth media and antibiotics

All liquid bacterial cultures were grown in 2×YT (ThermoFisher Scientific, Cat. No. BP9736), with the addition of additional chemicals and antibiotics as indicated, shaking at 250 rpm with a 1″ throw. Liquid bacterial cultures were grown in 2×YT supplemented with potassium phosphate (50 mM, pH 7) by adding 30.75 mL/L 1 M Potassium Phosphate Dibasic Solution (Sigma, Cat. No. P8584-4L) and 19.25 mL/L 1 M Potassium Phosphate Monobasic Solution (Sigma, Cat. No. P8709-4L) (hereafter referred to as 2×YTP). Overnight liquid cultures were grown in 50 mL Bio-Reaction Tubes (CellTreat Scientific Products, Cat. No. 229475) using 2×YTP media. Small scale protein expression experiments were performed using overnight cultures grown in 17 × 100 mm test tubes (Fisherbrand, Cat. No. 14-956-1J) to seed 25 mL media in 125 mL baffled culture flasks with 0.2 µM vented caps (Corning, Cat. No. 431405). Flasks were incubated at 37 °C using a Kuhner ISF-1-W Incubator Shaker using a 1″ throw at 250 rpm. The optical densities of the bacterial cultures were measured using 1.5 mL semi-micro cuvettes (Fisherbrand, Cat. No. 14955127) and an Implen P330 NanoPhotometer set to 600 nm and blanked with sterile growth media. Antibiotics used consisted of ampicillin (100 µg/mL), chloramphenicol (5 µg/mL) and zeocin (50 µg/mL), all purchased commercially. Cultures were supplemented using N6-(2-azidoethoxy)-carbonyl-l-lysine hydrochloride (hereinafter referred to as AzK) purchased from Synchem (Buffalo Grove, IL) and prepared to a 1 M stock solution in nuclease-free water.

### Construction of PylRS-expressing plasmid

The plasmid pSyn321 (pGEX-MbPylRS) was constructed by gene synthesis of a fragment corresponding to the sequence of the *M. barkeri* pyrrolysine aminoacyl tRNA synthetase (PylRS) and Gibson Assembly (Gibson Assembly Master Mix, New England Biolabs (NEB)) with a pGEX4T-1 plasmid (obtained from Genscript, Piscataway NJ) and contains the pBR322-type ColE1 origin of replication and ampicillin resistance. The assembly removed the glutathione S-transferase gene and multiple cloning site from pGEX4T-1 and place PylRS under the control of Ptac-lacO.

### Molecular assembly of expression plasmids containing the XY base pair

Insert fragments for Golden Gate assembly of the IL-2 coding region as well as the anticodon of the *M. mazei pylT* gene were generated by amplification of chemically synthesized oligonucleotides using both dTPT3TP and dNaMTP. Primers used in the PCR contained BsaI restriction enzyme recognition sites that when digested produce distinct overhangs which complement the cloning region of the destination plasmid, giving both directionality of the insert ligation as well as a “scar-less” final product. These insert template oligonucleotides were amplified at a concentration of 1 ng per 50 µL reaction using a mixture of OneTaq DNA Polymerase and Deep Vent Polymerase (NEB), 1× OneTaq buffer, 0.5× of SybrGreen, 3.0 mM MgSO4, 200 µM dNTPs, 100 µM of each unnatural nucleotide and 0.5 µM of each primer. SybrGreen was used to monitor amplification curves via qPCR. IL-2 XY inserts were amplified with the following thermocycling conditions (times denoted as mm:ss): [96 °C 1:00 | 15× (96 °C 0:15 | 50 °C 0:05 | 68 °C 4:00)], while the pylT tRNA inserts were amplified with slightly modified annealing temperature conditions: [| 5 × (94 °C 0:30 | 56 °C 0:05 | 68 °C 4:00), then | 10× (94 °C 0:30 | 62 °C 0:05 | 68 °C 4:00)]. Inserts were purified using the Zymo Clean and Concentrator Kit and quantitated using the Qubit DNA HS Assay Kit.

Golden Gate Assembly of the IL-2 expression constructs was done using 300 ng of destination plasmid with 25 ng of both *M. mazei* pylT gene and modified IL-2 coding region PCR-amplified Golden Gate inserts. The IL-2 Golden Gate entry vectors were linearized by PCR amplification using 0.02 U/µL Q5 DNA Polymerase with 200 µM dNTPs, 0.5× SYBR Green, 1× Q5 Reaction buffer, 2 ng of template per 50 µL reaction and 0.5 µM of primers which amplify from the IL-2 Golden Gate entry site using the following conditions: [98 °C 0:30 | 20× (98 °C 0:10 | 60 °C 0:10 | 72 °C 3:00)]. The resulting PCR was purified using ZymoClean PCR purification kit and the purified material was quantified using Qubit DNA HS Assay Kit. For ligation, 300 ng of vector DNA was combined with 25 ng of both pylT fragment and IL-2 inserts, 0.67 U/µL T4 DNA ligase, 0.67 U/µL BsaI-HF, 1× CutSmart buffer and 1 mM ATP in a 30 µL reaction volume thermal cycled under the following conditions: [37 °C 20:00 | 40× (37 °C 5:00 | 16 °C 5:00 | 22 °C 2:30) 37 °C 20:00 | 55 °C 15:00 | 80 °C 30:00]. Following the Golden Gate reaction, the mixture was incubated at 37 °C with T5 exonuclease and KpnI for 1 h to digest the unincorporated plasmid and insert fragments. Finally, the in vitro assembled unnatural plasmid stock was purified using a ZymoClean PCR purification kit and eluted in water.

### Expression strain generation

The *E. coli* BL21(DE3) variant YZ3^11^, which constitutively expresses a truncated version of the PtNTT2 nucleotide transporter from *P. tricornutum*, was obtained from the Romesberg lab at TSRI (San Diego). This strain was then transformed via CaCl_2_ chemical transformation with the plasmid pSyn321 (described above), plated on 2×YT agar containing 100 µg/mL ampicillin and 5 µg/mL chloramphenicol and incubated overnight at 37 °C. A single isolated colony was inoculated into a 2×YT medium containing 100 µg/mL ampicillin and 5 µg/mL chloramphenicol and grown overnight at 37 °C at 250 rpm shaking. The resulting culture, termed SYTX strain 37, was mixed 1:1 with 50% glycerol, frozen in liquid nitrogen and stored at −80 °C.

To generate the AzK-substituted IL-2 expression strains, an overnight culture of the parental strain SYTX169 was inoculated into 10 mL 2×YT media containing 5 µg/mL chloramphenicol, 5 µg/mL tetracycline and 50 mM potassium phosphate. The next day the culture was diluted back into an OD of 0.0025 using 50 mL fresh media and the culture was grown until an OD of 0.4. The cells were then chilled by swirling on ice for 5 min and centrifuged at 3000×*g* for 10 min 4 °C. The resulting cell pellet was washed twice using an equal volume of pre-chilled sterile deionized water using inversion and centrifuged. After the second wash, the cells were spun down a final time to remove excess water and resuspended in 300 µL of fresh pre-chilled sterile deionized water, in which 50 µL of the cells were used for electroporation with the following conditions: Voltage: 2.5 kV, Capacitor: 25 µF, Resistor: 200 Ω using a BioRad Gene Pulser II Electroporation System and a 2 mm electroporation cuvette. After electroporation, 1 mL of pre-warmed 2×YTP with 5 µg/mL chloramphenicol was added to the electroporated cells in which 44 µL of cells were added to 180 µL of 2×YTP + 5 µg/mL chloramphenicol with 187.5 µM dNaMTP and 47 µM dTPT3TP to rescue at a final concentration of 150 µM dNaMTP and 37.5 µM dTPT3TP. The cells were rescued at 37 °C shaking at 250 rpm for an hour and 100 µL of the rescued electroporated cells were inoculated into 3 mL of 2×YTP containing 5 µg/mL ampicillin, 5 µg/mL chloramphenicol, 50 µg/mL zeocin, 150 µM dNaMTP and 37.5 µM dTPT3TP and grown overnight. The next day the culture was expanded to 50 mL in the above media and monitored until reaching an OD of 1.0 in which the cells were chilled on ice and an equal volume of 50% glycerol was added and mixed. The mixture was aliquoted, frozen in liquid nitrogen and stored at −80 °C.

### Preparation of pegylated and un-pegylated IL-2 variants

Expression of IL-2 variants with AzK substitutions was performed in 2×YT medium (Thermo Fisher Scientific, Cat. No. BP9736) supplemented with 50 mM potassium phosphate, 100 µg/mL ampicillin, 5 µg/mL chloramphenicol, 50 µg/mL Zeocin, 150 µM dNaMTP and 37.5 µM dTPT3TP. Expression seeds were incubated overnight at 37 °C, diluted prior to reaching OD600nm of 1 before dilution in the same medium back to OD600nm of 0.05. Upon reaching OD600nm of ~0.8, cultures were pre-induced with 250 µM NaMTP, 25 µM TPT3TP and 10 mM AzK-HCl prepared in deionized water (Synchem, Buffalo Grove IL, product #36462). Approximately 15 min after pre-induction, cultures were induced with 1 mM IPTG and incubated for an additional 5 h. Cultures were collected by centrifugation at 16,000 × *g* for 20 min at 4 °C and pellets were stored at −80 °C until use.

Inclusion bodies were generated by the addition of 50 mL lysis buffer (1× PBS, Thermo Fisher Scientific, Cat. No. BP2940-4) containing protease inhibitors (Thermo Fisher Scientific, Cat. No. A32965) and 1× lysozyme (ThermoFisher Scientific, Cat. No. 89833), thawing pellets on ice and resuspending by pipetting. Resuspended pellets were lysed by passing twice through a microfluidizer (Dyhydrodymatics, model M110L). Lysed samples were centrifuged at 30,000 × *g* for 25 min at 4 °C before decanting and discarding the supernatant. Inclusion body pellets were solubilized by addition of 25 mL 6 M Guanidine-HCl in 100 mM Tris-HCl pH8, 20 mM imidazole per liter, pipetting until sample homogeneity before agitation for 15 min on a rotator. Samples were again centrifuged at 30,000 × *g* for 20 min at 4 °C and pellets were discarded.

To pre-equilibrate, Ni-NTA resin (ThermoFisher Scientific, Cat. No. 25216) was pre-washed with solubilization/wash buffer (6 M Guanidine-HCl, 100 mM Tris-HCl pH 8, 20 mM imidazole). The equilibrated resin was added to a clarified, solubilized sample in conical tubes and incubated for 1 h at 4 °C on a rotator. The resin was collected by centrifugation for 2 min at 250×*g* and the flow-through was removed. The resin was washed with 10–15CV of solubilization/wash buffer, transferred to a collection column and washed for an additional 10 column volumes of solubilization/wash buffer. The column was eluted using 3 CV of elution buffer (1x solubilization/wash buffer containing 500 mM imidazole). Eluates were dialyzed overnight in 12 L of 20 mM Tris-HCl pH 8/150 mM NaCl, then moved to 12 L of 20 mM Tris-HCl pH 8/50 mM NaCl for an additional 4 h. The resulting dialysate was centrifuged at 30,000×*g* 20 min 4 °C and supernatant was collected.

Samples were transferred to 50 mL conical tubes and 0.25 μL enterokinase (NEB cat# P8070L) was added per 5 µg of protein. Samples were mixed by inversion and incubated overnight at room temperature for enzymatic digestion. The pegylation reaction was initiated by the addition of 5 mM DBCO-mPEG (30 kDa Click Chemistry Tools, cat.# A121, 10 kDa cat #A119, or 5 kDa cat.#A118) stock to a final concentration of 50 µM and incubated overnight at 4 °C. Samples were concentrated using an Amicon-15 before loading onto HiLoad 16/600 Superdex 200 pg size-exclusion chromatography column (GE Healthcare) using an AKTA Pure system (GE Healthcare) pre-equilibrated and run in 1× PBS. Fractions of interest were analyzed by SDS-PAGE on a BioRad AnykD TGX Pre-cast gel followed by western blot using rabbit anti-IL-2 oligoclonal antibody (ThermoScientific, Cat. No. 710146). Peak fractions were pooled and adjusted to a final concentration of 4.5% acetonitrile 0.043% Trifluoroacetic acid. Samples were loaded onto a 3 mL RPC column (GE Healthcare) in 4.5% Acetonitrile/0.043% Trifluoroacetic acid and eluted using a gradient of buffer B (90% Acetonitrile/0.028% Trifluoroacetic acid). Fractions corresponding to the peak of interest were analyzed by SDS-PAGE and western blot as above, pooled and mixed 1:1 with dH_2_O and lyophilized using a Labconco FreeZone 4.5. Lyophilized samples were then resuspended in 50% acetonitrile 0.1% TFA and quantitated using a BSA standard curve with the BCA method to determine final concentration prior to final lyophilization as above. Samples were stored lyophilized at −80 °C until use.

### Discoverx PathHunter screening for pegylated IL-2 variants with “not alpha” pharmacology

Preminary cell-based pharmacological screening was performed under contract at DiscoverX (Fremont CA). In the Discoverx PathHunter® Cytokine Receptor Assay, one cytokine receptor chain is tagged with a small peptide epitope (ProLink (PK)) and the other chain is tagged with an Enzyme Acceptor (EA). Ligand binding induces dimerization of the two receptors, facilitating complementation of PK and EA fragments, which generates an active unit of beta-galactosidase and is detected using a chemiluminescent substrate.

PathHunter cell lines were expanded from freezer stocks and seeded in a total volume of 20 µL into white-walled, 384 well microplates and incubated for the appropriate time prior to testing. Cells were incubated with a sample to induce a response and intermediate dilution of sample stocks was performed to generate 5× sample in assay buffer, 5 µL of 5× sample was added to cells and incubated at 37 °C for 6–16 h. Vehicle concentration was 1%. The assay signal was generated through a single addition of 12.5 µL (50% v/v) of PathHunter Detection reagent cocktail, followed by a 1 h incubation at room temperature. Microplates were read following signal generation with a PerkinElmer EnvisionTM instrument for chemiluminescent signal detection. The compound activity was analyzed using the CBIS data analysis suite (ChemInnovation, CA). The percentage activity was calculated using the following formula: % Activity = 100% × (mean RLU of test sample − mean RLU of vehicle control)/(mean MAX RLU control ligand − mean RLU of vehicle control).

### Surface plasmon resonance (SPR)

The SPR studies herein were performed under contract by Biosensor Tools LLC (Salt Lake City UT). The molecular mass of IL-2 samples was assumed to be 15.5 kDa. The 50-µg samples were dissolved in 50 μL water to make stock solutions of 64.5 μM. The Fc-tagged human IL-2 receptor subunits (extracellular domains) were purchased from Sino Biological (hIL-2 Ra-Fc Sino 10165-H02H, hIL-2 Rb-Fc Sino 10696-H02H). Recombinant human IL-2 (hIL-2) was purchased from Thermo Scientific (Cat. No. PHC0021) and prepared at a stock concentration of 64.5 μM.

Using Biacore 2000 optical biosensor, the Fc-IL-2 receptor subunits were immobilized to Protein A-coated CM4 sensor chip to densities of ~160–170 RU and equilibrated with running buffer (10 mM HEPES, 150 mM NaCl, 0.005% Tween-20, 0.1 mg/mL BSA, pH 7.4). Binding studies were performed at 25 °C. When necessary, the Protein A surfaces were regenerated with 150 mM phosphoric acid between binding cycles. The test articles were tested in duplicate, in two-fold dilution series starting at 2 µM. The responses from IL-2 Rβ surfaces were fit to a 1:1 interaction model to obtain binding parameters using Scrubber (v2.0c).

### Flow cytometry assay for pSTAT5 induction in primary human PBMC

The studies reported herein complied with all relevant ethical regulations for animal testing and research.

Primary human PBMC potency studies using flow cytometry were performed under contract by PrimityBio (Fremont CA) or internally. PrimityBio purchased human blood samples through an IRB with Stanford University (Reg#: 5136 (eprotocol 13942) and 6208 (eprotocol 38735)). Internal blood samples collection from anonymous healthy donors has been approved by the Institutional Review Board of The Scripps Research Institute (#177065). Leukocyte reduction systems (LRS) were purchased from Cell IDX (San Diego, CA). All subjects provided informed consent. The studies were performed following the guidelines of the World Medical Association’s Declaration of Helsinki.

Primary human lymphocyte populations from 3–6 (Peg size study (Supplementary Fig. 5) and pSTAT5 potency assay (Fig. 2), respectively) independent healthy donors were sourced fresh and prepared from leucocyte reduction system (LRS) collected from anonymous healthy donors. Samples were treated in triplicate with either hIL-2 (Aldesleukin) or THOR-707 in 3-fold dilution series starting with a top concentration of 30 µg/mL. After a brief incubation, samples were fixed and stained with antibodies to detect pSTAT5, a marker of upstream engagement and activation of IL-2 receptor signaling complexes and a panel of antibodies that stain surface markers specific for lymphocyte populations of interest to follow pSTAT5 formation in specific T cell and natural killer (NK) cell subpopulations. The half-maximal effective concentration (EC50) levels for pSTAT5 signaling potency were measured in primary CD8^+^ T, NK and Treg cell subpopulations.

Lyophilized compound from the manufacturer was reconstituted according to the manufacturer’s recommendations. Briefly, the compound was resuspended in 0.1 M acetic acid to 2 mg/mL at room temperature and mixed by flicking. Upon complete solubilization (by visual inspection for clarity), the compound was aliquoted into small aliquots, frozen in liquid nitrogen and stored at −80 °C. The rhIL-2 (Gibco #PHC0023 or #PHC0027) or THOR-707 samples were stored frozen in 0.1 M acetic acid prior to use in the assays. Stated concentrations throughout this report refer to the protein component of the pegylated compound to facilitate direct activity comparisons between pegylated and non-pegylated compounds.

Human leukocyte reduction system (LRS) cones were received the same day as drawn from healthy donors. The LRS chambers were drained into a 50 mL conical tube and the volume was adjusted to 20 mL using 1× PBS. Then 90 µL of the stock was aliquoted into the relevant wells of a 96-well plate. The plate is warmed to 37 °C for 15 min prior to the addition of the compound.

Compounds were diluted in PBS and the rhIL-2 was diluted using PBS + 0.1% BSA to create 10× stocks. The 10× IL-2 stock concentration was µg/mL and the THOR-707 stocks were between 6 and 300 µg/mL, depending on the experiment. The 10X stocks were diluted in successive 5-fold dilutions to create a 10-point dose titration. The top dose of the IL-2 was µg/mL and the THOR-707 stock was between 6 and 300 µg/mL depending on the experiment. 10 µL of each stock was added to 90 µL of blood samples to achieve a final top dose for IL-2 of 500 ng/mL and 0.6–30 µg/mL for THOR-707.

To stimulate, 10 µL of the dose titration outlined above was added to 90 µL of blood sample pre-equilibrated to 37 °C. The samples were incubated at 37 °C for 45 min. At the end of the incubation period, the red blood cells were lysed and the cells were fixed simultaneously as follows: 100 µL cells were transferred to 900 µL of BD Lyse/Fix Buffer (Beckton Dickinson, Cat# 558049) and mixed immediately. The BD Lyse/Fix was prepared by diluting the stock 1:5 with cell culture water just prior to addition. Samples were incubated 10 min at room temperature, then centrifuged at 450 × *g* for 5 min to pellet cells. Pelleted cells were washed with PBS + 0.5% BSA and stored at −80 °C until analysis.

Cells were thawed at room temperature and TruStain FcX (Biolegend 422302) was added with 15-fold dilution. After 5 min at room temperature, cell surface antibodies were added. After 20 min at room temperature, cells were washed two times with PBS + 0.5% BSA and permeabilized by adding 10 volumes of Methanol (Fisher Chemical, Cat. A412-4) to one volume of cells or Perm Buffer III (Becton Dickinson, Cat. 558050). The permeabilization was incubated for 10+ min at 4 °C, washed with PBS, with PBS + 0.5% BSA and Fc block was added as above before the addition of the following post-permeabilization staining panel. The stained cells were incubated for 1 h at room temperature, washed twice with PBS with 0.5% BSA and prepared for flow cytometric analysis. The antibody details used in these procedures are provided in Supplementary Table 11 below.

### Flow cytometry and data analysis instrumentation

Samples were run on Becton Dickinson Fortessa, LSRII, or ThermoFisher Attune NxT instrument. The 96-well plates containing the stained samples were run at less than 8000 cells/s using the 96-well high throughput sampler. Data were collected and exported as.fcs files to a network drive. Once the data was exported it was uploaded to the google cloud using the CellEngine browser-based flow cytometry analysis program (PrimityBio). Within CellEngine, the data was compensated to account for spillover of the fluorophores and the fcs files are annotated. The fcs files are then gated on singlets using FSC-A by FSC-H to exclude any aggregates or doublets. Within this gate, the cells are gated on mid to high forward scatter (FSC-A) and side scatter (SSC-A) to exclude the red blood cells, debris and granulocytes (Lymphocyte gate). The T cells are then gated as the CD3^+^, CD56/16 negative population. The NK cells are identified as the CD3 negative, CD56/16 high population. The T cells are then divided into CD4^+^ T and CD8^+^ T cells. The Tregs are then gated from the CD4^+^ T cells as the CD25hi × C127lo population. Alternatively, fcs files were analyzed in FlowJo v10 following similar gating strategies. A graphical description of the gating strategies outlined above is shown in Supplementary Fig. 16 below.

The median fluorescence intensity (MFI) for each of the cell population, donor and compound treatment was calculated from the signal in the channel detecting phosphorylated STAT5 using CellEngine software. The statistics were analyzed using Spotfire or GraphPad Prism. Within Spotfire, the data was plotted on a log scale for the compound doses and a linear scale for the MFI readings. These data were fit using a 4-parameter logistic regression equation. The EC50 was calculated as the inflection point of the curve.

### Quantitative analysis of THOR-707 by ELISA

Bioanalysis of plasma samples was performed using a human IL-2 ELISA assay (Abcam, cat. #10056). Concentrations of THOR-707 in plasma tumor and spleen samples and the internal standard were determined using the ELISA assay. The software SoftMax Pro (v7.1) was used to capture and analyze ELISA data and Phoenix WinNonlin 8.1 was used for PK analysis. PK data analysis was performed at NW Solutions (Seattle, WA).

### Tumor cell lines

The B16-F10 cell line was purchased from the American Tissue Type Collection (ATCC, Manassas, VA) by Crown Biosciences. Briefly, the cells were grown in DMEM (ATCC) supplemented with 10% fetal bovine serum and penicillin/streptomycin. All cells were maintained at 37 °C in a humidified incubator, with the atmosphere equilibrated at 10% CO_2_ and 90% air.

### Subcutaneous xenograft

C57BL6 mice were housed at 72 ± 5 °F, ambient humidity and 12 h light: dark cycle. Animal welfare for this study complies with the U.S. Department of Agriculture’s Animal Welfare Act (9 CFR Parts 1, 2 and 3) as applicable. All experimental data management and reporting procedures were in strict accordance with applicable Crown Bioscience, Inc. Guidelines and Standard Operating Procedures.

B16-F10 cells were washed in serum-free media, counted and resuspended in cold serum-free media at a concentration of 50,000 viable cells/0.1 mL. All cells were injected subcutaneously in the flank of the C57BL6 mouse. Tumor measurements were collected three times per week for the duration of the study using digital calipers. To calculate volume, a prolate ellipsoid model was used to estimate tumor volume (mm^3^) from two-dimensional tumor measurements:

$${{{{{\rm{Tumor}}}}}}\; {{{{{\rm{volume}}}}}}\; ({{{{{{\rm{mm}}}}}}}^{3})=({{{{{\rm{length}}}}}}\times {{{{{{\rm{width}}}}}}}^{2})\div2.$$

Assuming unit density, volume was converted to weight (i.e., 1 mm^3^ = 1 mg).

In the B16-F10 tumor PK/PD study, mice were randomized for treatment when the average tumor volume reaches 80–100 mm^3^. In the B16-F10 efficacy study, mice were randomized at 50 mm^3^ for the treatment. The starting of dosing day is designated as day 0.

### Blood collection and processing for PK and immune cell profiling

After a single intravenous injection of mice with THOR-707, terminal blood samples were collected by cardiac puncture following CO_2_ euthanasia. For the study in naive mice, blood was collected at 13 time points (0.03, 0.17, 0.5, 1, 2, 4, 8, 12, 24, 48, 72, 96 and 120 h) post-dose, sacrificing 3 mice per time point. For the study in tumor-bearing mice, blood was collected at 9-time points (day 0 [2, 8, 12 h], 1, 2, 3, 5, 7 and 10 days) post-dose, sacrificing 4 mice per time point. 200 µL of plasma was collected for PK analysis.

For immune cell phenotyping, 400 µL of blood were treated with 20 volumes (8 mL) of pre-warmed Lyse/Fix Buffer (BD Phosflow™, catalog #558049) and incubated for 10 min at 37 °C. Cells were spun for 8 min at 500 × *g* and washed twice with 5.0 mL of flow cytometry buffer (BD, catalog #561550). Lysed and fixed blood cells were first blocked with TruStain FcX (BioLegend, 101320) at room temperature for 5 min before the surface marker staining using the antibody cocktail shown below in the table. cells were permeabilized with 4 °C pre-cooled methanol at room temperature for 10 min, followed by the intracellular staining with the antibody cocktail listed in the 2nd table. The events were acquired with Becton Dickinson Fortessa. The CD8^+^ T cells are then gated as the CD3^+^ CD8^+^ T population. The NK cells are identified as the CD3^−^ NK1.1^−^ population. The Tregs were then gated from the CD4^+^ T cells as the CD25^+^ FoxP3^+^ population. The memory CD8 cells were identified as CD44hi CD8^+^ population. Data are represented as cell population as a percent of its parental or grandparental cell population. The antibody panel used to profile the immune cell phenotyping from whole blood is shown in Supplementary Table 12 below.

### Tissue collection and processing

In the B16-F10 tumor PK/PD study, after a single intravenous bolus administration of THOR-707, the tumors and spleens were harvested at day 0 (2, 8, 12 h), 1, 2, 3, 5, 7 and 10, following CO_2_ euthanasia. The tumor was separated into two halves, one half was weighed and frozen down in liquid nitrogen for tumor PK analysis.

Half of the tumors for flow cytometry analysis were processed right after collection. MACS mouse tumor dissociation kit (Miltenyi Biotec) was used to process tumor samples into single cells for flow cytometry analysis. Briefly, tumor samples were minced into small pieces, followed by mechanical and enzymatic digestion with Gentle MASC (Miltenyi Biotec).

Mouse splenocytes were dissociated by homogenizing spleens via straining using the plunger end of a syringe, then washed with 1× PBS, followed by red blood cell lysis with 1× red blood cell lysis buffer (Invitrogen, catalog #00-4333-57).

### Tumor and spleen lysate preparation for pk analysis

Frozen tumor or spleen samples were homogenized with the lysis buffer (made by dissolving 1 tablet of protease inhibitor [SIGMA, catalog #4693159001] in 10 mL 1× PBS). Every 0.1 g tissue was mixed with 0.4 mL of that buffer. To each tissue collection tube, a 5 mm stainless steel bead was added (Qiagen, catalog #69989) prior to homogenization with Tissue Lyser II (Qiagen) at 20 Hz for 20 s. Following homogenization, the tissue tumor lysate was spun down and the supernatant was collected for THOR-707 PK analysis.

### In vivo immune cell phenotyping in B16-F10 tumor PK/PD study

Lysed and fixed whole-blood samples from the B16-F10 tumor PK/PD study were analyzed with the same method as described above in the section “Blood collection and processing for PK and immune cell profiling”. The antibody panel used to profile the immune cell phenotyping for tumor and spleen samples in the B16-F10 tumor PK/PD study is shown in Supplementary Table 13 below.

### IL-2 (P65K) protein production for crystallization

Crystallization studies were performed under contract by Proteros (Munich Germany). For crystallization studies, the His-IL-2 (P65K, C125S) protein variant was expressed in *E. coli* BL21(DE3) using 2×YT medium supplemented with 50 mM Potassium Phosphate and 50 µg/mL Zeocin. The pellet from 3 L above culture was homogenized with Ultra Turrax in 150 mL 1× PBS pH 7.4 with DNAse1, 4 Roche Complete Tabs and lysozyme (1 mg/mL). After incubation on ice for 30 min, the sample was lysed using sonication 10 × 30 s. After centrifugation at 33,000×*g* (25 min, 4 °C) the resulting pellet was homogenized with Ultra Turrax and centrifuged again. The final pellet was solubilized in 75 mL 6 M Guanidinine-HCl, 100 mM Tris-HCl pH 8 and 20 mM Imidazole.

The denatured His-IL-2 (P65K, C125S) protein was captured using 20 mL Ni-Sepharose using gravity flow, washed with 15 column volumes of 6 M Guanidine-HCl, 100 mM Tris-HCl pH 8, 20 mM imidazole before elution in 3 column volumes of 6 M Guanidine-HCl, 100 mM Tris-HCl pH 8, 500 mM imidazole. Eluate solution was dialyzed against 10 L 20 mM Tris/HCl pH 8, 150 mM NaCl overnight, centrifuged and dialyzed for another 4 h against 10 L 20 mM Tris/HCl pH 8, 50 mM NaCl.

The resulting protein was digested with enterokinase (100 U/mg). After 20 h digestion time, the resulting sample was concentrated to 1.5 mg/mL and purified on Superdex S-200 26/60 run at 2 mL/min in 20 mM Tris-HCl pH8, 50 mM NaCl collecting 3 mL fractions. Fractions containing the peak were pooled and concentrated to 37 mg/mL (UV). In sum, the procedure yielded homogenous protein with a purity greater than 95% as judged from Coomassie-stained SDS-PAGE.

### Crystallization

The purified protein was used in crystallization trials employing a standard screen with approximately 1200 different conditions. Conditions initially obtained were optimized by systematically varying parameters critically influencing crystallization, such as temperature, protein concentration, drop ratio and others. These conditions were also refined by systematically varying pH or precipitant concentrations. Crystal growth was improved by streak seeding with a diluted solution of crushed crystals in the reservoir solution. The final crystallization condition was: 1.75 M (NH_4_)_2_SO_4_, 0.10 M HEPES/NaOH pH = 7.75 and the protein was used at a concentration of 37 mg/mL.

### Data collection and processing

Crystals were flash-frozen in liquid nitrogen and measured at a temperature of 100 K. The cryo buffer consisted of 20% (v/v) ethylene glycol and 80% (v/v) reservoir solution. The X-ray diffraction data were collected at the SWISS LIGHT SOURCE (SLS, Villigen, Switzerland) using cryogenic conditions.

The crystals belong to space group P 4_3_ 2_1_ 2. Data were processed using the programs autoPROC, XDS and autoPROC, AIMLESS.

### Structure modeling and refinement

The phase information necessary to determine and analyze the structure was obtained by molecular replacement. A previously solved structure of IL-2 was used as a search model. Subsequent model building and refinement were performed according to standard protocols with COOT and the software package CCP4, respectively. For the calculation of the free R-factor, a measure to cross-validate the correctness of the final model, about 9.6% of measured reflections were excluded from the refinement procedure (Supplementary Table 5).

TLS refinement (using REFMAC5, CCP4) was performed, which resulted in lower R-factors and higher quality of the electron density map.

The water model was built with the “Find waters”-algorithm of COOT by putting water molecules in peaks of the 2*F*_o_ − *F*_c_ map contoured at 3.0 followed by refinement with REFMAC5 and checking all waters with the validation tool of COOT. The criteria for the list of suspicious waters were: B-factor greater 80 Å^2^, 2*F*_o_ − *F*_c_ map <1.2 σ, distance to closest contact <2.3 Å or more than 3.5 Å. The suspicious water molecules were checked manually.

The Ramachandran plot of the final model shows 94.3% of all residues in the most favored region, 5.7% in the additionally allowed region and 0.0% in the generously allowed region. No residues are found in the disallowed region (Supplementary Table 5). Statistics of the final structure and the refinement process are listed in Supplementary Table 5.

### Reporting summary

Further information on research design is available in the Nature Research Reporting Summary linked to this article.

## Supplementary information

Supplementary Information

Reporting Summary

## Data Availability

The structure factors and coordinates for the rhIL-2 (P65K) structure have been deposited into the Protein Data Bank under the accession number 7M2G. The coordinates for rhIL-2 bound to the heterotrimeric IL-2 receptor complex structure used for structural alignments is publicly available (2ERJ). Source data are provided with this paper.

## Source data

Source Data

## Code availability

The data sets generated during and/or analyzed during the studies reported herein do not make use of custom code or mathematical algorithms central to the conclusions.
