# Supplementary material for: An engineered IL-2 reprogrammed for anti-tumor therapy using a semi-synthetic organism
Source: Nat Commun. 2021 Aug 9;12:4785. doi: 10.1038/s41467-021-24987-9 (PMC8352909; doi:10.1038/s41467-021-24987-9)
Supplement: Supplementary file 1 — Supplementary Information [file 41467_2021_24987_MOESM1_ESM.pdf]

Supplementary Information

Ptacin et al. 2021

An engineered IL-2 reprogrammed for anti-tumor therapy using a semi-synthetic organism

Supplementary Table 1. PEG IL-2 candidate screening using DiscoverX PathHunter recombinant cell-based screening for differential IL-2Rβγ vs αβγ agonist activity

Site	βγ EC50 (nM)	αβγ EC50 (nM)	βγ/αβγ ratio
rhIL-2	2.23	0.14	16
K35	6.75	0.15	45
R38	4.16	0.17	25
T41	6.37	0.05	130
F42	6.09	0.52	12
K43	9.84	0.13	75
Y45	9.06	0.11	83
E62	3	1.5	2
E62*	7.46	2.84	3
P65*	23.8	4.44	5
E68*	7.7	0.09	86
V69*	9.99	0.08	121

* 30kDa mPEG

Discoverx PathHunter assay for IL-2 receptor agonist activity. Human U2OS cells engineered to express the IL-2 receptor β and γ subunits fused to portions of a split enzyme system (EA: β -galactosidase fragment 1; PK: β -galactosidase fragment 2). IL-2 engagement of the β subunit elicits recruitment of the γ subunit and receptor dimerization. Receptor dimerization stimulates reconstitution of the β-galactosidase activity, which is read using chemiluminescence.

Shown are potency statistics for rhIL-2 and IL-2 compounds pegylated with 10 kDa (or 30 kDa where indicated) mPEG modifications in the DiscoverX PathHunter IL-2 receptor βγ (second column) and αβγ (third column) assays. The βγ: αβγ EC50 ratios for each test compound are listed in the right column. For a molecule with complete reduction in IL-2Rα binding, the βγ: αβγ EC50 ratio is predicted to be 1, assuming identical receptor subunit stoichiometry between cell lines.

Supplementary Figure 1. THOR-707 was identified using recombinant cell-based screening as a potent IL-2Rβγ agonist with reduced IL-2Rα engagement

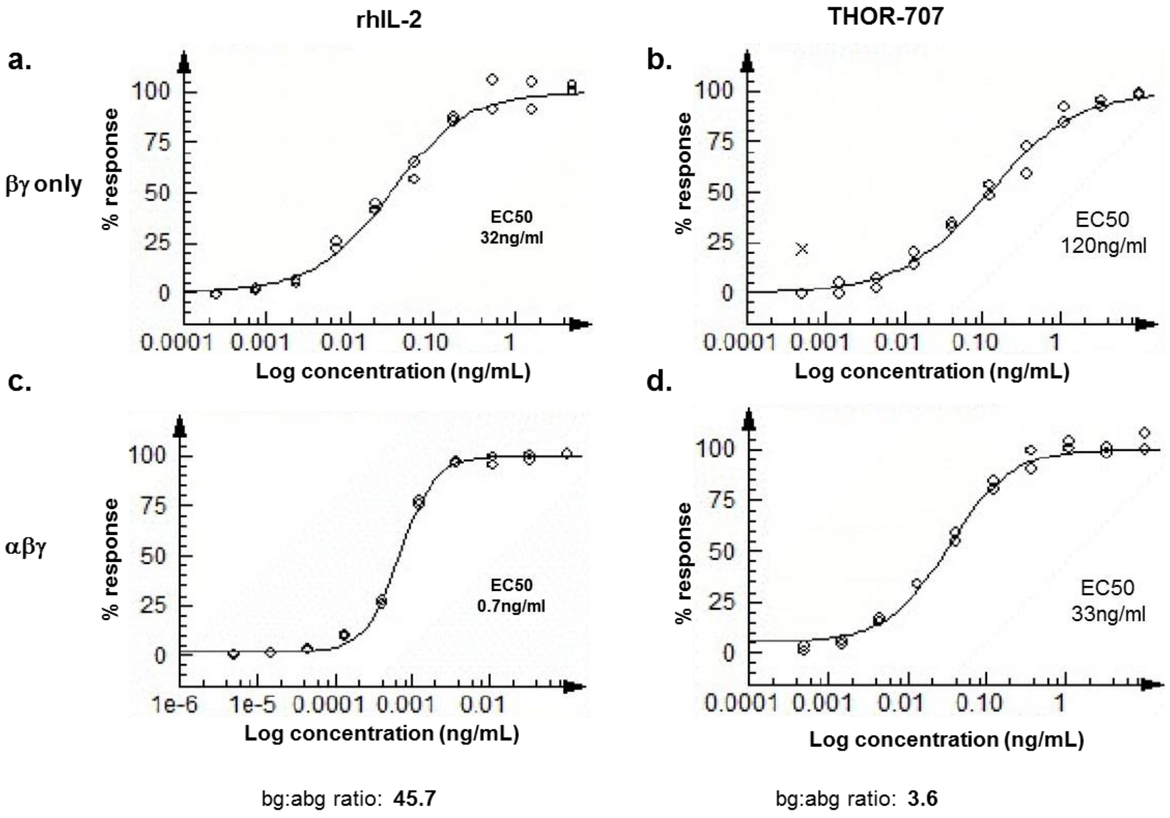

Supplementary Figure 1. THOR-707 was identified using recombinant cell-based screening as a potent IL-2Rβγ agonist with reduced IL-2Rα engagement. Recombinant human IL-2 (left column) or THOR-707 (right column) were analyzed in the DiscoverX PathHunter IL-2 receptor βγ (top row) and αβγ (bottom row) assays. Concentration response curves are shown for rhIL-2 in the IL-2Rβγ (A) and αβγ assays (B). Concentration response curves are shown for THOR-707 IL-2Rβγ (C) and αβγ assays (D). Assays were run in duplicate. Response (Y-axis, percent signal normalized to Aldesleukin) is plotted in percent units, and dose (X-axis, ng/mL) is plotted on a log10 scale. The βγ: αβγ EC50 ratios for each test compound are listed at the bottom of the plots. For a molecule with complete reduction in IL-2Rα binding, the βγ: αβγ EC50 ratio is predicted to be 1, assuming identical receptor subunit stoichiometry between cell lines. Source data are provided as a Source Data file.

Supplementary Figure 2. Biochemical characterization of THOR-707 interactions with IL-2 α and β receptor subunits using SPR demonstrate “not alpha” signature of THOR-707 and modest PEG size dependence to IL-2 Rβ affinity.

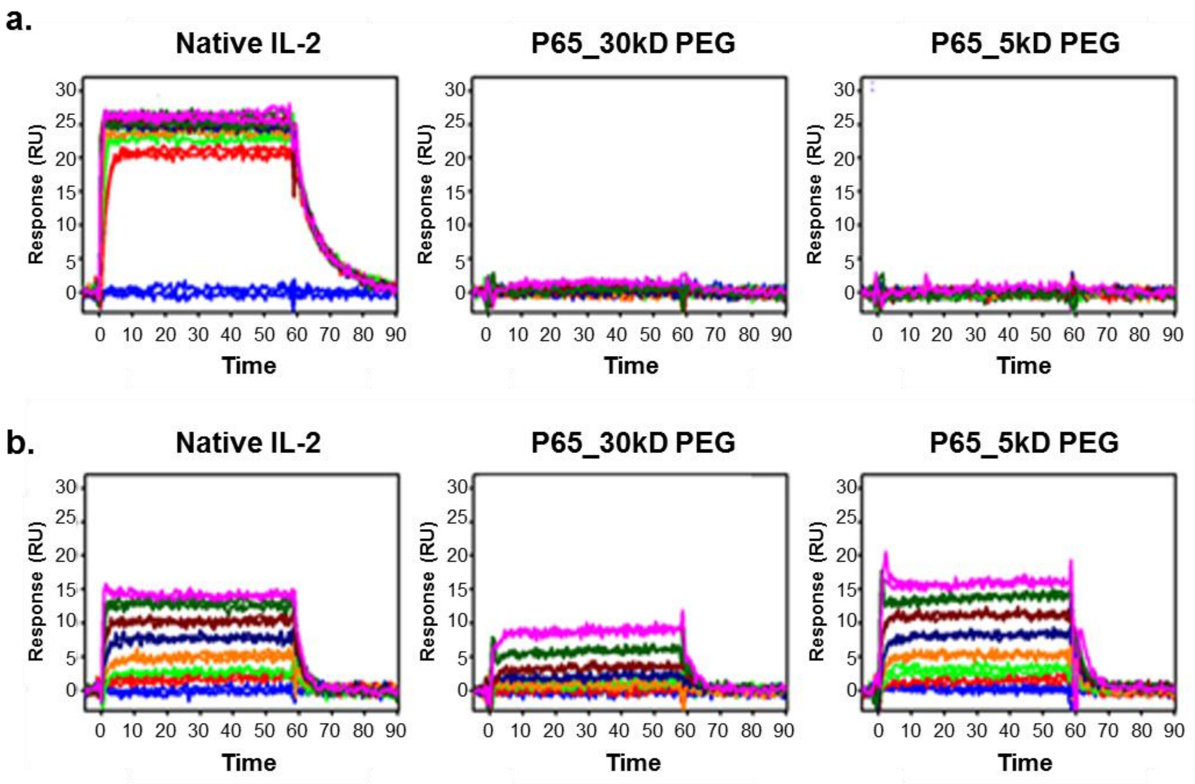

Supplementary Figure 2. . Biochemical characterization of THOR-707 interactions with IL-2 α and β receptor subunits using SPR demonstrate “not alpha” signature of THOR-707 and modest PEG size dependence to IL-2 Rβ affinity. Human IL-2 Rα (A) and β (B) extracellular domains were immobilized on the surface of a SPR sensor and probed in duplicate with two-fold dilution series starting at 2μM of either rhIL-2 (left column), THOR-707 (30kDa mPEG, middle column), or THOR-707 (5kDa mPEG, right column). Test samples were injected for 60 s to allow measurement of association, followed by buffer only (wash) for 30 s to measure dissociation kinetics. Response units (RU, Y-axis) are plotted versus time (s, X-axis). Colors correspond to test concentration as shown in Figure 2b (inset).

Supplementary Table 2. Kinetic parameters for rhIL-2 and THOR-707 interactions with human IL-2 Rα and Rβ subunit extracellular domain surfaces

IL-2Rα		$k_a (M^{-1}s^{-1})$	$k_d (s^{-1})$	$K_D (\mu M)$
	rhIL-2	$4.5 \pm 0.3 \times 10^7$	0.410 ± 0.01	0.009 ± 0.002
	THOR-707 (30kDa mPEG)	114 ± 36	0.018 ± 0.008	158 ± 21
	THOR-707 (5kDa mPEG)	797 ± 226	0.033 ± 0.004	42 ± 7
IL-2 Rβ		$k_a (M^{-1}s^{-1})$	$k_d (s^{-1})$	$K_D (\mu M)$
	rhIL-2	$1.3 \pm 0.2 \times 10^6$	0.185 ± 0.009	0.15 ± 0.05
	THOR-707 (30kDa mPEG)	$1.8 \pm 0.2 \times 10^5$	0.370 ± 0.01	2.09 ± 0.09
	THOR-707 (5kDa mPEG)	$9.0 \pm 0.4 \times 10^5$	0.270 ± 0.01	0.31 ± 0.02

Values reported represent the mean and standard deviation from a fit of data sets reported. The values for the association rate of rhIL-2 to human IL-2 Rα are rapid and mass transport limited under these conditions, leading to a higher measurement error. However, because the association and dissociation rates are highly correlated the ratio of the kinetic parameters for highly mass transport limited data still provides an accurate value for the equilibrium dissociation constant K_D.

Supplementary Table 3. Dose-response curve fit statistics for rhIL-2 and THOR-707 stimulation of pSTAT5 in LRS-based flow cytometry assay for potency

A. Data for rhIL-2

Treatment	Donor	Population	min	max	Hill	LoggedX50	EC50 pg/ml	y50	r2
IL-2	1	CD8	1481.1	6664.9	1.0	4.1	12792.8	4073.0	0.99
IL-2	2	CD8	1457.6	8442.8	1.1	3.8	7065.6	4950.2	0.95
IL-2	3	CD8	1657.9	8190.8	1.1	4.0	9198	4924.4	0.96
IL-2	4	CD8	972.3	6393.3	1.2	3.6	3915.8	3682.8	0.99
IL-2	5	CD8	687.2	6772.7	1.0	3.8	6257.7	3729.9	0.99
IL-2	6	CD8	688.0	6797.6	1.3	3.8	5664.1	3742.8	0.99
IL-2	1	NK cells	886.5	5966.6	1.1	3.6	3950.3	3426.6	1.00
IL-2	2	NK cells	520.3	6316.4	1.3	3.6	4225.5	3418.4	0.96
IL-2	3	NK cells	452.8	5090.2	1.1	3.8	5659.2	2771.5	0.98
IL-2	4	NK cells	440.8	4077.9	1.5	3.4	2688.9	2259.3	0.98
IL-2	5	NK cells	623.2	4575.1	1.9	3.1	1261.7	2599.1	0.99
IL-2	6	NK cells	552.4	5450.5	1.6	3.3	2139.5	3001.4	0.99
IL-2	1	Treg	1516.5	17201.7	1.4	1.6	38.7	9359.1	0.98
IL-2	2	Treg	618.1	16776.3	1.0	1.6	40.1	8697.2	0.85
IL-2	3	Treg	1326.6	11103.4	1.3	1.9	73.6	6215.0	0.94
IL-2	4	Treg	414.0	11785.6	1.2	1.4	24.9	6099.8	0.97
IL-2	5	Treg	1080.6	13965.4	1.7	1.2	14.9	7523.0	0.97
IL-2	6	Treg	1023.9	13095.5	1.7	1.2	14.6	7059.7	0.96

B. Data for THOR-707

Treatment	Donor	Population	min	max	Hill	LoggedX50	EC50 pg/ml	y50	r2
THOR-707	1	CD8	1373.5	6562.3	1.9	5.0	103916.8	3967.9	0.99
THOR-707	2	CD8	1315.2	8269.2	1.4	5.0	92984.7	4792.2	0.98
THOR-707	3	CD8	1474.5	7986.2	1.5	5.0	102482.4	4730.3	0.97
THOR-707	4	CD8	948.3	7123.3	1.6	4.8	65221.1	4035.8	0.98
THOR-707	5	CD8	818.1	6061.9	2.2	4.9	88682.5	3440.0	0.99
THOR-707	6	CD8	628.0	7841.6	1.4	5.0	103687.3	4234.8	0.99
THOR-707	1	NK cells	857.2	5425.1	2.0	4.6	42315.9	3141.1	0.99
THOR-707	2	NK cells	468.1	5532.0	1.5	4.7	46090.4	3000.0	0.98
THOR-707	3	NK cells	423.4	4664.8	1.6	4.8	56589.5	2544.1	0.98

THOR-707	4	NK cells	414.9	4581.0	1.5	4.6	35937	2498.0	0.98
THOR-707	5	NK cells	701.5	4832.0	1.8	4.5	31720.1	2766.8	0.97
THOR-707	6	NK cells	587.4	6120.0	1.7	4.6	38020.1	3353.7	0.98
THOR-707	1	Treg	1468.2	12885.8	5.3	4.9	88033.2	7177.0	0.96
THOR-707	2	Treg	1037.1	12884.8	1.5	4.8	56952.8	6960.9	0.96
THOR-707	3	Treg	1067.7	10006.9	1.5	5.0	99608.6	5537.3	0.99
THOR-707	4	Treg	758.6	10946.8	1.3	4.9	73033	5852.7	0.98
THOR-707	5	Treg	1097.5	12856.4	2.4	4.8	57880.9	6976.9	0.97
THOR-707	6	Treg	939.6	12502.1	1.8	4.8	60658.8	6720.8	0.99

Supplementary Table 4. Data collection and processing statistics for IL-2 (P65K) structure determination

**Variant**	**IL-2 (P65K)**
X-ray source	PXII/X10SA (SLS[1])
Wavelength [Å]	1.0000
Detector	EIGER
Temperature [K]	100
Space group	P 4_3 2_1 2
Cell: a; b; c; [Å]	73.50; 73.50; 76.97
α; β; γ; [◦]	90.0; 90.0; 90.0
Resolution [Å]	1.79 (1.82-1.79)
Unique reflections	20517 (983)
Multiplicity	16.1 (15.7)
Completeness [%]	100.0 (100.0)
R_{pim} [%][6]	1.3 (48.3)
R_{sym} [%][3]	4.8 (186.6)
R_{meas} [%][4]	5.0 (192.9)
CC1/2 [%]	99.90 (70.10)
Mean(I)/sd[5]	27.8 (1.6)

[1] SWISS LIGHT SOURCE (SLS, Villigen, Switzerland)

[2] values in parenthesis refer to the highest resolution bin.

[3] Equation 1

$$Rsym = \frac{\sum_h \sum_i^{n_h} |\hat{I}_h - I_{h,i}|}{\sum_h \sum_i^{n_h} I_{h,i}} \quad \text{with} \quad \hat{I}_h = \frac{1}{n_h} \sum_i^{n_h} I_{h,i}$$

where $I_{h,i}$ is the intensity value of the *i*th measurement of *h*

[4] Equation 2

$$Rmeas = \frac{\sum_h \sqrt{\frac{n_h}{n_h - 1}} \sum_i^{n_h} |\hat{I}_h - I_{h,i}|}{\sum_h \sum_i^{n_h} I_{h,i}} \quad \text{with} \quad \hat{I}_h = \frac{1}{n_h} \sum_i^{n_h} I_{h,i}$$

where $I_{h,i}$ is the intensity value of the *i*th measurement of *h*

[5] calculated from independent reflections

[6] Equation 3

Precision-indicating $Rpim = \frac{\sum_h \sqrt{1/(N-1)} |I_{hl} - <I_h>|}{\sum_h <I_h>}$

Supplementary Table 5. Refinement statistics for IL-2 (P65K)[1]

Variant	IL-2 (P65K)
Resolution [Å]	53.16-1.79
Number of reflections (working /test)	18553 / 1964
R_{cryst} [%]	19.2
R_{free}[%][2]	22.8
Total number of atoms:	
Protein	1128
Water	101
Sulfate	5
Glycerol	6
Deviation from ideal geometry: [3]	
Bond lengths [Å]	0.015
Bond angles [°]	1.71
Bonded B's [$Å^2$] [4]	6.3
Ramachandran plot: [5]	
Most favoured regions [%]	94.3
Additional allowed regions [%]	5.7
Generously allowed regions [%]	0.0
Disallowed regions [%]	0.0

[1] Values as defined in REFMAC5, without sigma cut-off
[2] Test-set contains 9.6% of measured reflections
[3] Root mean square deviations from geometric target values
[4] Calculated with MOLEMAN
[5] Calculated with PROCHECK

Supplementary Figure 3. Structure of THOR-707 P65K demonstrates that substitution of proline 65 for lysine does not affect IL-2 tertiary structure or positioning of residues contacting IL-2 Rα or β subunits

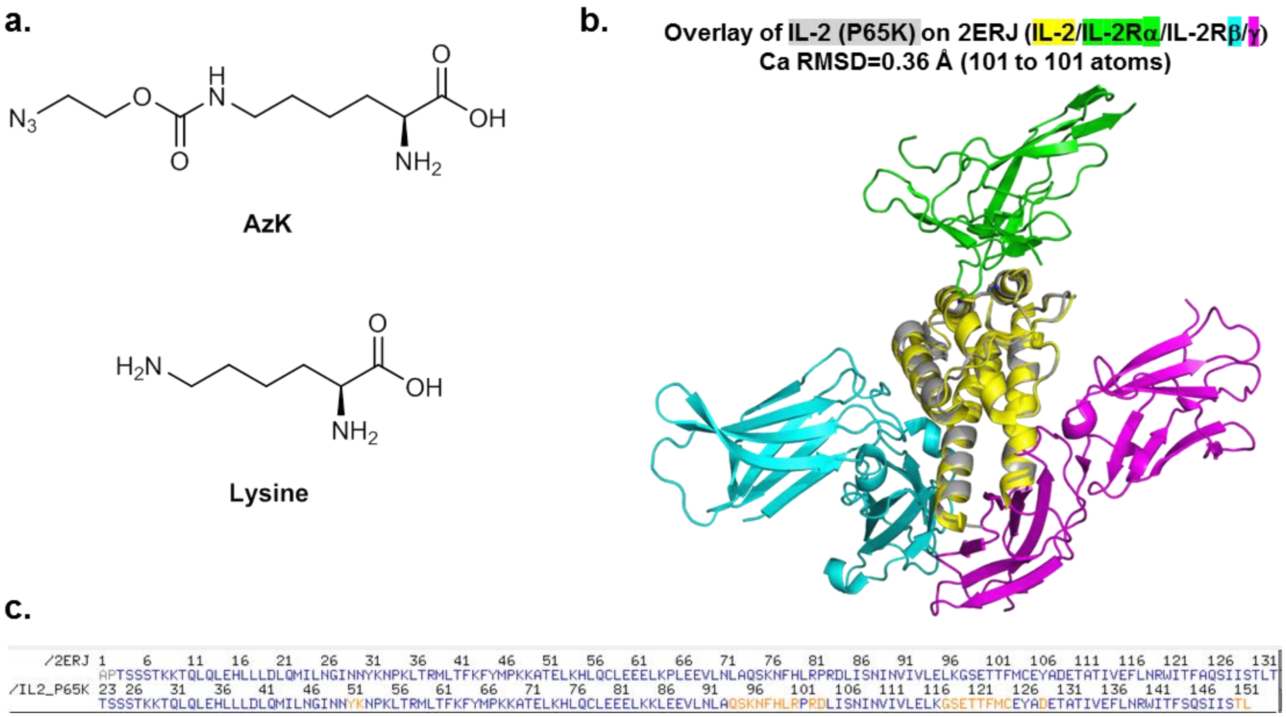

Supplementary Figure 3. Structure of THOR-707 P65K demonstrates that substitution of proline 65 for lysine does not affect IL-2 tertiary structure or positioning of residues contacting Rα or β subunits. A. Structural similarity of AzK (top) and lysine (bottom) residues. B. Substitution of position 65 with lysine does not affect the overall structure and folding of THOR-707 compared to rhIL-2. The crystal structure of IL-2(P65K) was aligned onto 2ERJ (DOI: 10.2210/pdb2ERJ/ pdb) using PyMOL. The RMSD is 0.36 Å over 101 atoms as PyMOL aligned Cα atoms. C. Alignment of rhIL-2 (2ERJ) and IL-2 P65K sequences is shown. Residues corresponding to flexible loop regions that were automatically excluded from the Pymol analysis are highlighted in orange.

Supplementary Figure 4. Impact of P65K mutation on the environment of position 65 of IL-2.

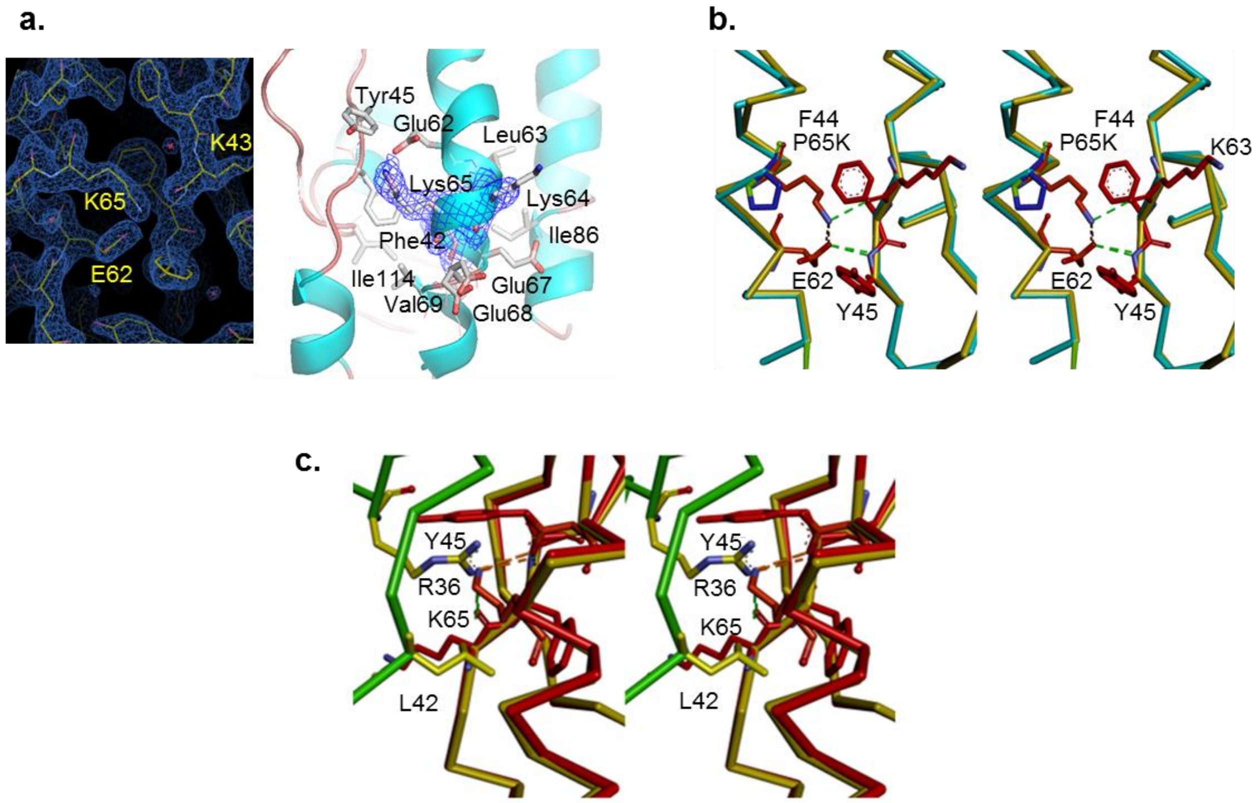

Supplementary Figure 4. Impact of P65K mutation on the environment of position 65 of IL-2.

A. The mutation site P65 is located in the center of helix B where the proline residue supports a significant bend of the helix. The electron density of the neighboring four-helix bundle core of the protein is strong and especially Lys65 is completely defined by electron density. The side chain of Lys65 and neighboring protein side chains are shown as stick model, colored according to the chemical atom type superimposed with the refined 2Fo-Fc electron density map contoured at 1.0 angstrom. The residue K65 makes direct contacts with E62 in this structure.

B. Stereo view of the overlay of IL-2 (P65K) (gold) and apo-IL-2 (PDB: 1M47, DOI: 10.1073/pnas.252756299, blue). The overlay of the IL-2-P65K mutant and an the 1M47 apo-IL-2 structure shows that mutation of proline to lysine does not influence the position of backbone and side chain atoms in the near vicinity within experimental error. Some larger deviations are observed toward the loop structures which may be attributed to the inherent flexibility of the loops.

C. Stereo view close-up showing the interface between IL-2 (red: IL-2(P65K); gold IL-2 wild-type) and IL-2Rα (green). Lys65 is predicted to collide with R36 of IL-2Rα.The crystal structure of the complex between IL-2 and its receptors revealed the interaction between the components IL-2Rα, IL-2Rβ and IL-2Rβ/γ in detail (PDB: 2ERJ (DOI: 10.2210/pdb2ERJ/pdb). The interface of IL-2 and IL-2 Rα includes a region around position 65 which is composed of hydrophobic interactions but comprises also a buried salt-bridge between Glu62 of IL-2 and Arg36 of IL-2

Rα. The overlay between IL-2/receptor complex and IL-2(P65K) shows that the lysine residue introduced in THOR-707 is predicted to collide with Arg36 of IL-2Rα. In particular, the epsilon amine group of Lys65 superimposes almost exactly with a guanidinium nitrogen of Arg36. None of the alternative side-chain conformations of Lys65 is predicted to alleviate these clashes. It is therefore predicted that without main-chain alterations in the region from Arg35 to Met44 of IL-2Rα the introduction of Lys65 in IL-2 will interfere with binding to IL-2 Rα.

Supplementary Figure 5. Reduction in THOR-707 engagement of IL-2 Rα is mediated largely by AzK substitution at P65, pegylation contributes to minor and non-specific reduction in potency.

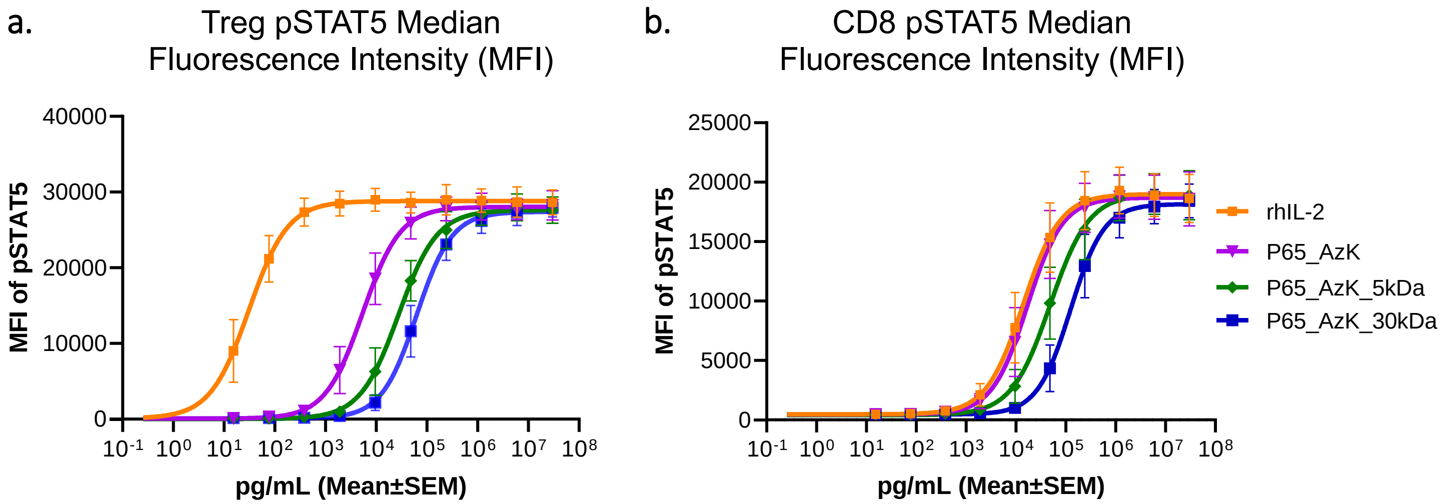

c.

Compound	Treg EC50 (pg/mL)	CD8+ T cell EC50 (pg/mL)
rhIL-2	31	14,048
P65_AzK	5446	16,908
P65_AzK_5KDa	27,710	48,904
P65_AzK_PEG 30KDa	62,838	125,442

Supplementary Figure 5. Reduction in THOR-707 engagement of IL-2 Rα is mediated largely by AzK substitution at P65, pegylation contributes to minor and non-specific reduction in potency.

Fresh human PBMC samples from 3 healthy donors were stimulated with concentration series of rhIL-2 or THOR-707 variants with P65AzK (unmodified), P65AzK clicked to a 5kDa mPEG, or THOR-707 (30kDa mPEG substituent). Treated cell populations were analyzed using multi-color flow cytometry to detect and quantify pSTAT5 activation in (A) Treg, and (B) CD8+ T cells. The plots shown represent the average pSTAT5 signal fit to a baseline restrained 4 parameter variable slope least-squares fit, with error bars representing SEM. (C) Table shows the extracted EC50 values for the curves in (A) and (B). The specific reduction in potency in Tregs vs CD8+ T cells for P65AzK compound demonstrates that substitution of P65 with AzK mediates the majority of the “not alpha” pharmacology of THOR-707, while PEGylation results in a PEG-size dependent and non-specific reduction in receptor potency. (n=3 independent donor samples) Source data are provided as a Source Data file.

Supplementary Figure 6. THOR-707 demonstrates increased exposure relative to rhIL-2 in mouse.

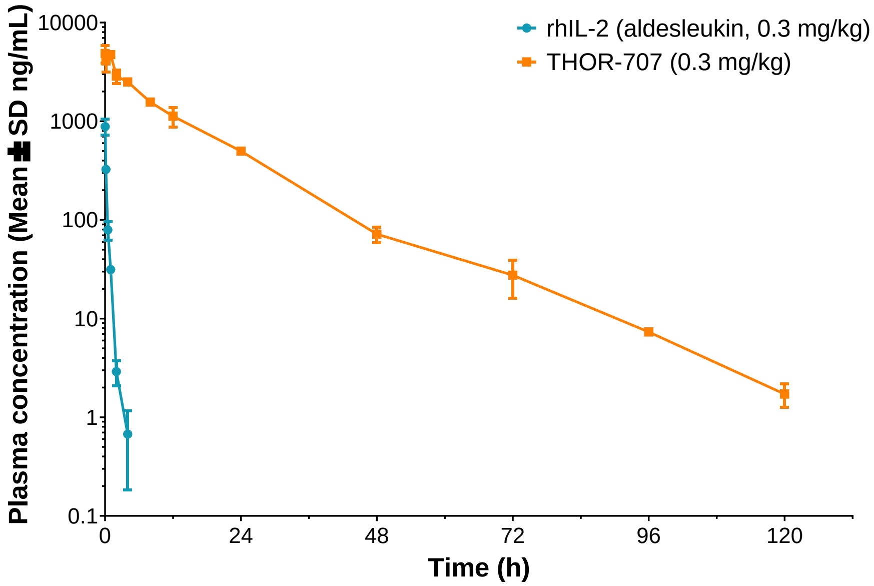

Supplementary Figure 6. THOR-707 shows increased exposure relative to rhIL-2 in mouse.

Mean (±SEM) plasma concentration vs. time profiles following a single intravenous bolus dose of rhIL-2 (blue) or THOR-707 (red) to female C57BL/6 mice (the dose refers to IL-2 polypeptide amount). The plot shows mean ± standard error (SEM), n= 3 mice per time point. Source data are provided as a Source Data file.

Supplementary Table 6. Plasma PK Parameters Following a Single intravenous Dose of rhIL-2 or THOR-707 in Naïve and Tumor-bearing Mice

Parameter	Units	Aldesleukin 0.3 mg/kg	THOR-707 Doses (mg/kg)		
			0.3	1	3
			naive	B16-F10 tumor bearing	B16-F10 tumor bearing
T_{max}	h	0.030	0.030	2.00*	2.00*
C_{max}	ng/mL	884	4,870	12,900	40,000
$AUC_{0\text{-}t}$	h•ng/mL	229	45,600	174,000	656,000
R^2	---	0.900	0.992	0.983	0.974
AUC_{INF}	h•ng/mL	230	45,600	174,000	656,000
$t_{1/2}$	h	0.573	13.3	14.2	12.6
CL	mL/h/kg	1,300	6.58	5.74	4.57
V_{ss}	mL/kg	390	82.4	68.4	62.1

* First time point measured.

Note: R^2 is the goodness-of-fit parameter for the terminal phase of each concentration vs. time profile.

Supplementary Figure 7. THOR-707 induced a transient increase in pSTAT5 in NK cells in naïve and B16-F10 tumor-bearing mice

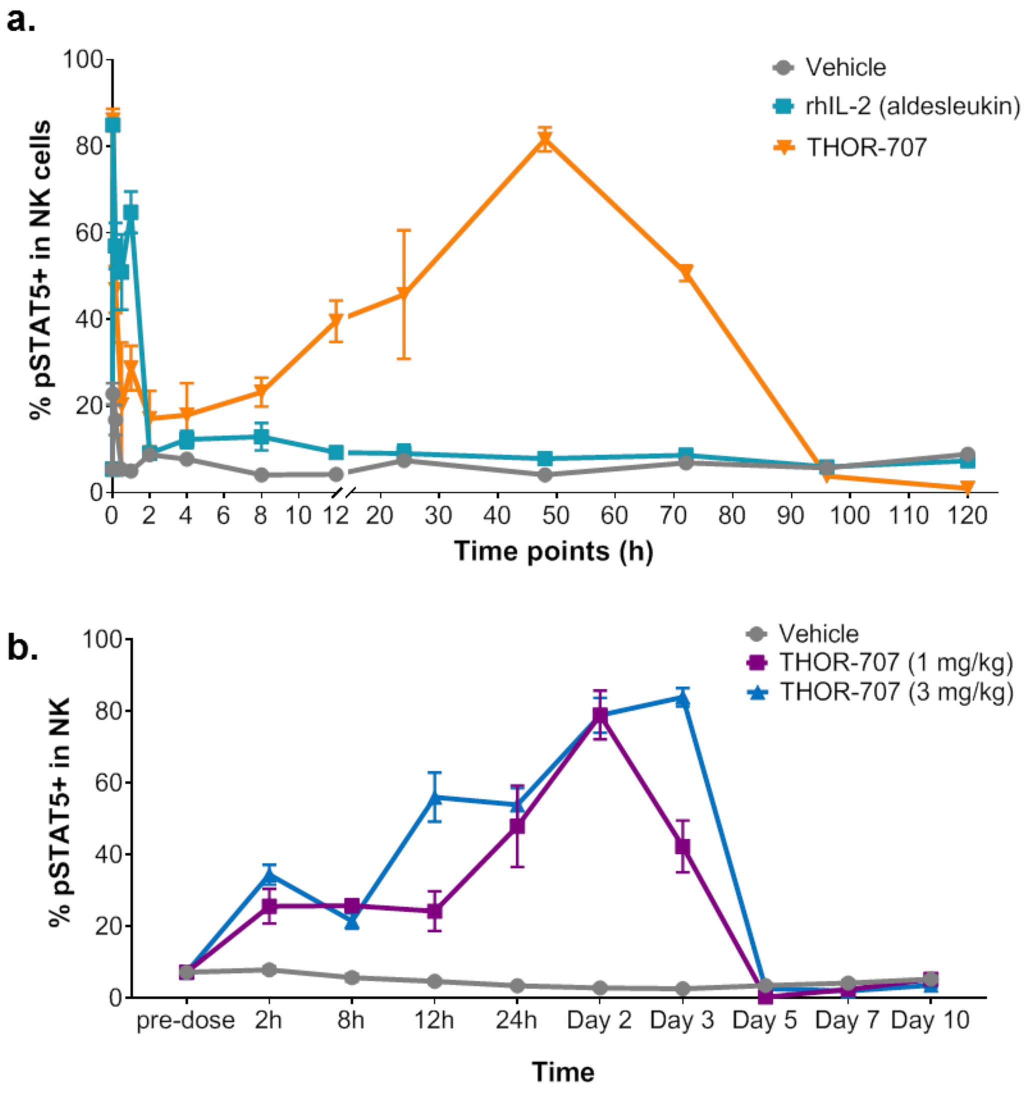

Supplementary Figure 7. THOR-707 induced a transient increase in pSTAT5 in NK cells in naïve and B16-F10 tumor-bearing mice. Percentage of pSTAT5+ cells in peripheral blood NK cells following administration of a single intravenous bolus dose of: (A) 0.3 mg/kg of THOR-707 or Aldesleukin to naïve mice, and (B) 1 or 3 mg/kg of THOR-707 to tumor-bearing mice. Blood was drawn via cardiac puncture at the indicated time points and immune cell populations were assessed by FACS. Each data point represents an average from replicates at each time point ± SEM. (n=3 animals) Source data are provided as a Source Data file.

Supplementary Figure 8. THOR-707 induced a sustained increase in pSTAT5 in CD4+ Tregs

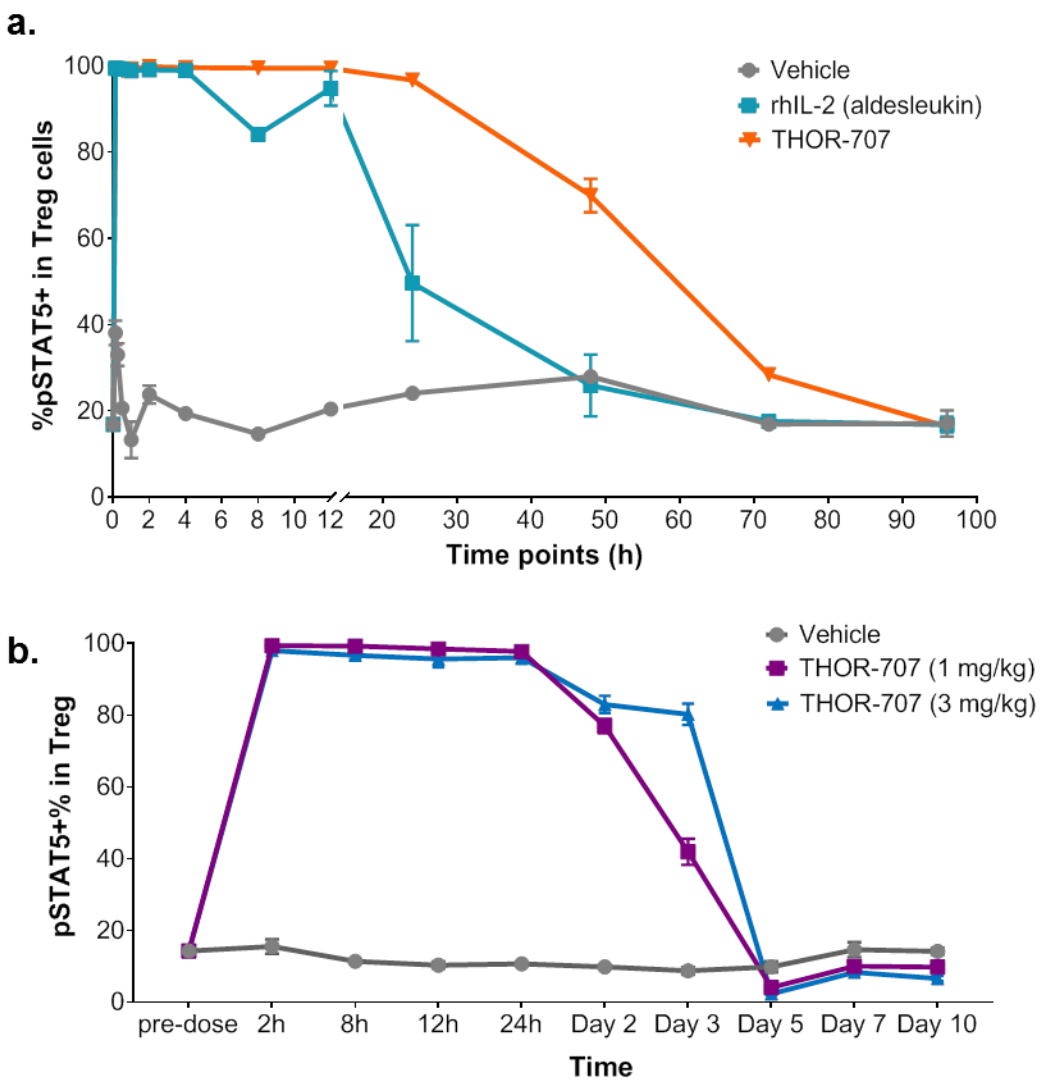

Supplementary Figure 8. THOR-707 induced a sustained increase in pSTAT5 in CD4+ Tregs

Percentage of pSTAT5+ cells in peripheral blood CD4+ Treg cells in following administration of a single intravenous bolus dose of: (A) 0.3 mg/kg of THOR-707 or Aldesleukin to naïve mice, and (B) 1 or 3 mg/kg of THOR-707 to tumor-bearing mice. Blood was drawn via cardiac puncture at the indicated time points and immune cell populations were assessed by FACS. Each data point represents an average replicates at each time point ± SEM. (n=3 animals) Source data are provided as a Source Data file.

Supplementary Figure 9. THOR-707 induced the expression of Ki67 in peripheral blood CD8+ T, NK, and Treg cells

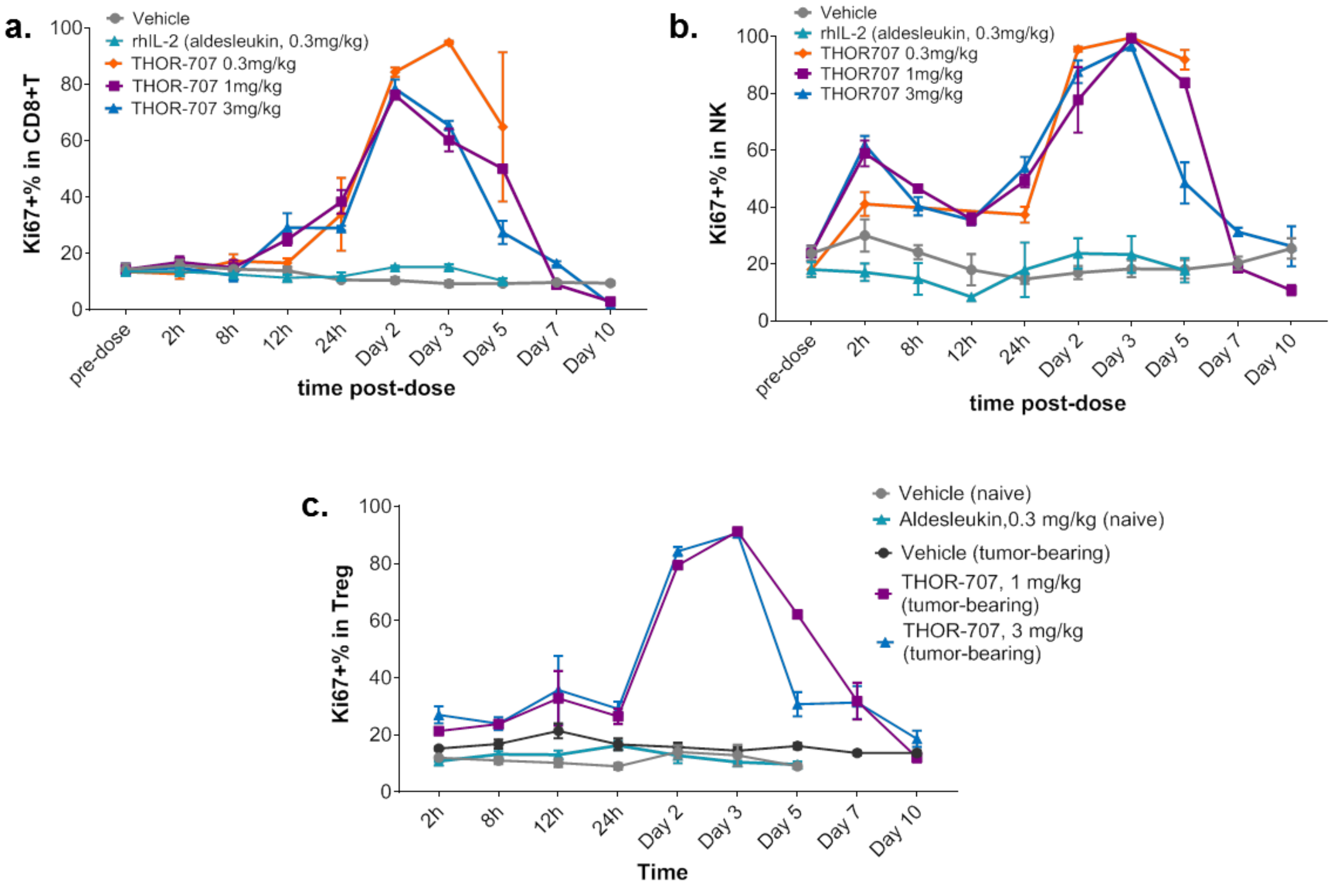

Supplementary Figure 9. THOR-707 induced the expression of Ki67 in peripheral blood CD8+ T, NK, and Treg cells. Percentage of Ki67+ cells in peripheral blood: (A) CD8+ T cells, (B) NK cells, and (C) Treg cells following administration of a single intravenous bolus dose of either 0.3 mg/kg of THOR-707 or Aldesleukin to naïve mice or 1 or 3 mg/kg of THOR-707 to tumor-bearing (note in panel C, due to insufficient events during FACS in many time points, the THOR-707 at 0.3 mg/kg group is not included in the figure). Blood was drawn via cardiac puncture at the indicated time points and immune cell populations were assessed by FACS. Each data point represents an average from replicates at each time point ± SEM. (n=3 animals) Source data are provided as a Source Data file.

Supplementary Figure 10. THOR-707 induced the expansion of peripheral blood CD8+ memory T cells in naïve C57BL/6 mice

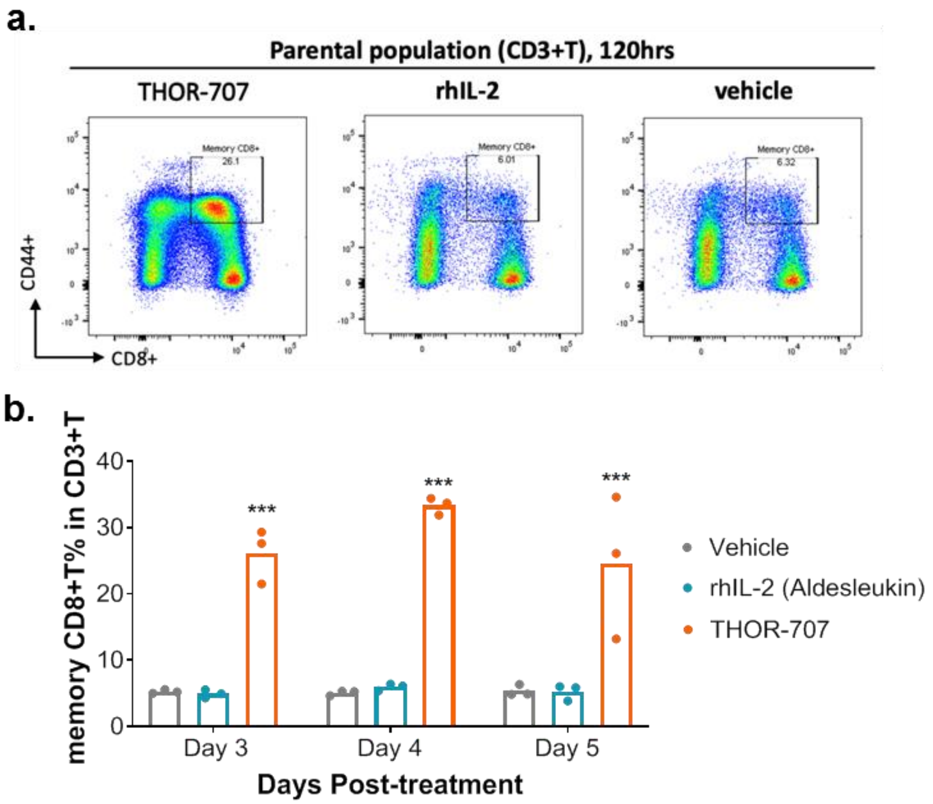

Supplementary Figure 10. THOR-707 induced the expansion of peripheral blood CD8+ memory T cells in naïve C57BL/6 mice.

A. Plots showing CD3+ cells analysed for CD44+ (Y axis) and CD8+ (x axis) after treatment with THOR-707 (left), rhIL-2 (middle), or vehicle (right). The memory CD8+ T cell population is indicated and quantitated in the square. B. Percentage of CD8+ memory T cells within the CD3+ T cell population up to 5 days post-dose of a single intravenous bolus dose of THOR-707 or Aldesleukin at 0.3.mg/kg. Each data point represents an average from replicates at each time point ± SEM. *** p<0.001 vs. vehicle or Aldesleukin, analyzed using unpaired t test. P values <0.05 were considered significant (***are defined with P values <0.001) (n=3 animals) Source data are provided as a Source Data file.

Supplementary Figure 11. THOR-707 induced the expansion of peripheral blood CD8+ memory T cells in naïve C57BL/6 mice. A single THOR-707 drives improved CD8+ T and NK cell expansion compared to multiple, frequent Aldesleukin doses, but results in lower serum IL-5 levels. (n=3 animals) Source data are provided as a Source Data file.

a. Experimental dosing levels and schedule. Due to the short half-life and limited exposure of rhIL-2, Aldesleukin was dosed BID x 3 to achieve pharmacodynamic response of expansion of CD8+ T cells and NK cells (shown in B).

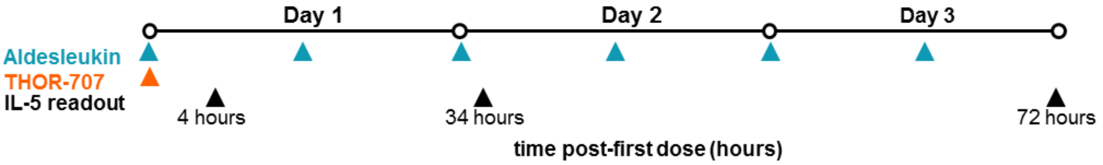

b. Pharmacodynamic effects of each compound and dose observed at 72 hours post first dose are shown below, with the percent of total peripheral cells (singlets) that were CD8+ T cells (left) or NK cells (right) shown. Bars represent the mean of the experimental replicates, and points show the values for each individual. Compared to Aldesleukin, THOR-707 administration resulted in similar or increased expansion of CD8+ T and NK cells at all doses.

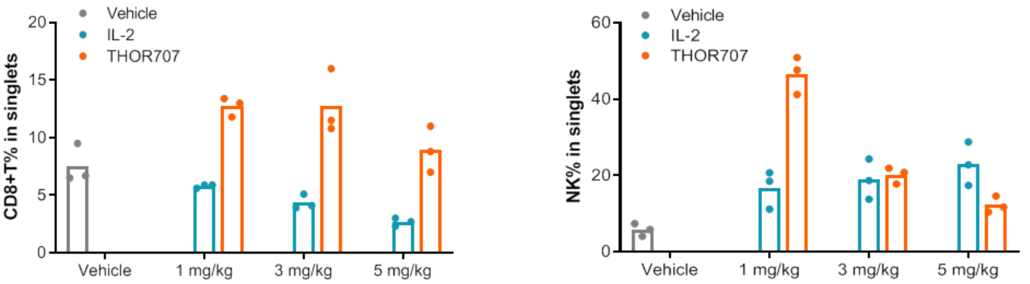

c. Serum IL-5 levels (pg/mL) are shown for each dose of Aldesleukin (green) and THOR-707 (orange) at 4hr, 34hr, and 72hr post first-dose. Bars represent the mean serum IL-5 levels (pg/ml) in 3 individual animals, and data points show the values for each individual. Despite the similar or increased expansion of CD8+ T cells and NK cells, administration of THOR-707 resulted in lower IL-5 serum levels at each condition and time.

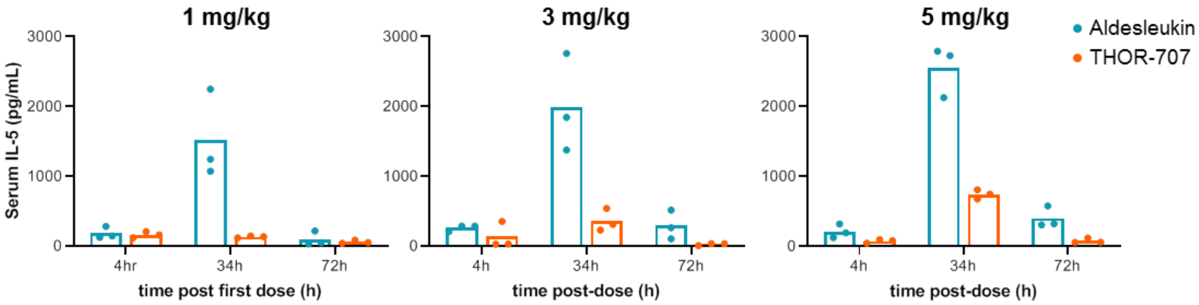

d. Serum IL-5 levels (pg/mL) are shown for each dose of Aldesleukin (green) and THOR-707 (orange) at 4hr, 34hr, and 72hr post first-dose. Bars represent the mean serum IL-5 levels (pg/ml) in 3 individual animals, and data points show the values for each individual. Despite the similar or increased levels of expansion of CD8+ T cells and NK cells, administration of THOR-707 resulted in lower IL-5 serum levels at each condition and time.

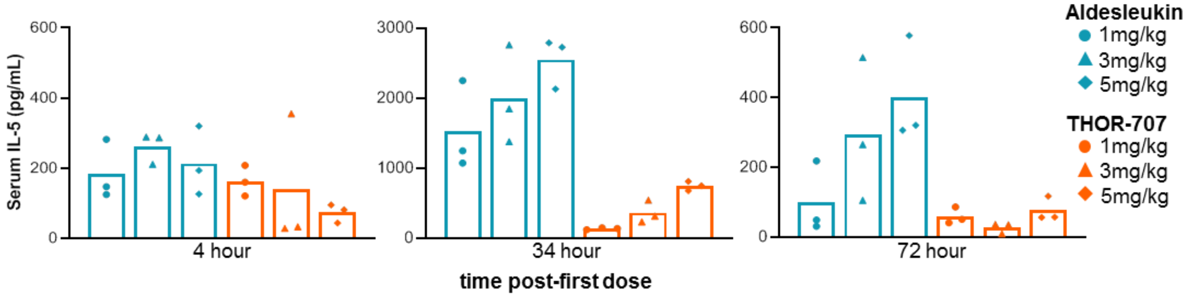

Supplementary Table 7. A single THOR-707 drives improved CD8+ T and NK cell expansion compared to multiple, frequent Aldesleukin doses, but results in lower plasma IL-5 levels

Data table showing the raw values from individual animals for serum IL-5 levels (pg/mL) for each dose of Aldesleukin and THOR-707 at 4hr, 34hr, and 72hr.

	4 hours time point								
Dose (mg/kg)	Vehicle			Aldesleukin			THOR-707		
0	36.7	12.9	16.9						
1				124.6	281.8	146.6	120.3	159.6	207.7
3				286.5	288.4	210.6	32.2	27.8	354.7
5				193.4	126.1	319.8	94.6	43.4	81.0
Dose (mg/kg)	34 hours time point								
	Vehicle			Aldesleukin			THOR-707		
0	12.0	1.9	5.0						
1				1072.4	2249.1	1245.8	119.2	145.6	135.7
3				2759.5	1845.2	1377.2	312.8	230.3	540.9
5				2789.3	2128.1	2727.4	676.9	809.0	746.4
Dose (mg/kg)	72 hours time point								
	Vehicle			Aldesleukin			THOR-707		
0	2.6	0.8	1.8						
1				30.6	218.1	48.9	50.7	40.9	86.5
3				515.4	265.0	105.2	35.2	9.3	36.3
5				578.2	320.7	306.3	57.3	56.4	117.1

Supplementary Figure 12. THOR-707 shows enhanced retention in tumor tissue relative to plasma and spleen tissue in B16-F10 tumor bearing mice.

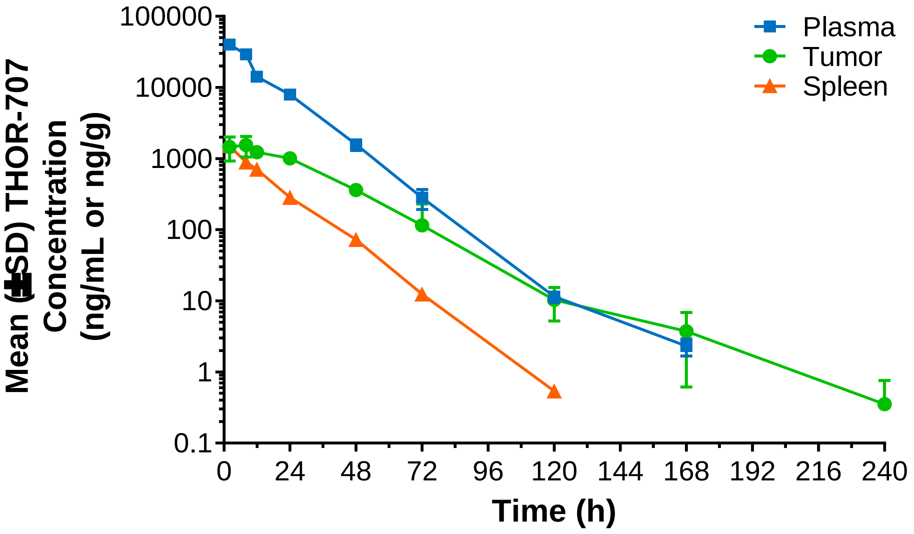

Supplementary Figure 12. THOR-707 shows enhanced retention tumor tissue relative to plasma and spleen tissue in B16-F10 tumor bearing mice. Plasma, tumor, and spleen PK following administration of a single bolus intravenous dose of THOR-707 at 3 mg/kg. The plot shows mean ± SD, n = 3 mice per time point. Errors for some points are small and obscured by the marker. Source data are provided as a Source Data file

Supplementary Table 8. THOR-707 plasma, tumor, and spleen PK Parameters in B16-F10 tumor-bearing C57BL/6 Mice

Parameter	THOR-707 Dose					
	1 mg/kg			3 mg/kg		
	Plasma	Tumor	Spleen	Plasma	Tumor	Spleen
T_{max} (h)	2.00	8	2.00	2.00	8	2.00
C_{max} (ng/mL)	12,900	463	466	40,000	1,550	1,510
$t_{1/2}$ (h)	14.20	25.80	10.80	12.60	24.60	10.20
AUC_{0-t} (h•ng/mL)	174,000	16,800	7,040	656,000	55,200	23,500
R^2	0.983	1.0	0.991	0.974	0.988	0.999
AUC_{INF} (h•ng/mL)	174,000	16,800	7,040	656,000	55,200	23,500

Supplementary Figure 13. THOR-707 strongly induced the CD8+ T cell expansion and increased the CD8+ T/Treg ratio in the spleens of tumor-bearing mice

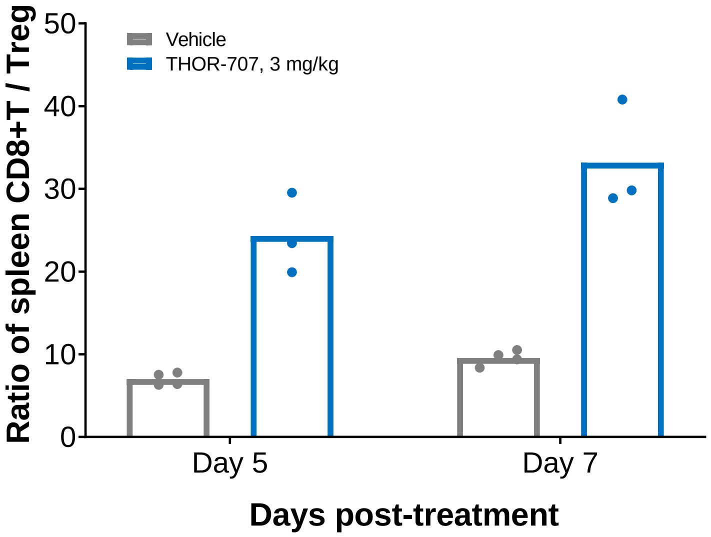

Supplementary Figure 13. THOR-707 strongly induced the CD8+ T cell expansion and increased the CD8+ T/Treg ratio in the spleens of tumor-bearing mice. The ratio of CD8+ T over CD4+ Treg cells at 5 and 7 days following treatment with a single 3 mg/kg intravenous bolus dose of THOR-707. Each data point represents an average from replicates at each time point ± SEM (N=4 vehicle, N=3 THOR-707). Source data are provided as a Source Data file.

Supplementary Figure 14. THOR-707 induced the expansion of peripheral blood CD8+ T cells

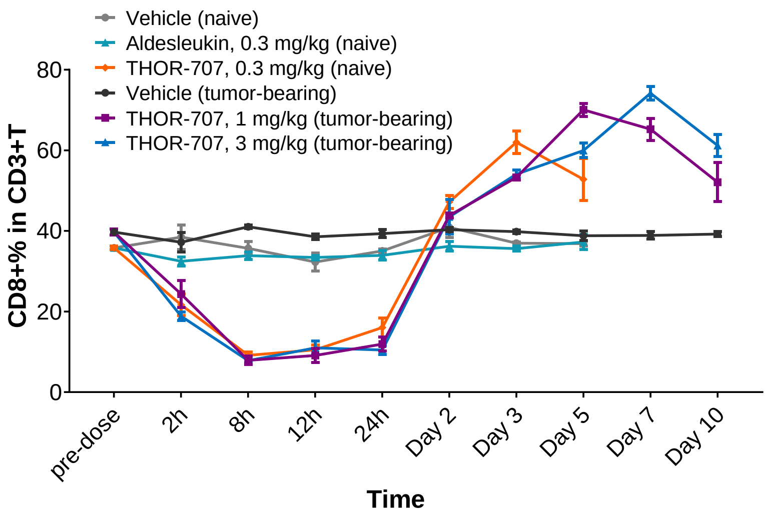

Supplementary Figure 14. THOR-707 induced the expansion of peripheral blood CD8+ T cells

Percentage of CD8+ cells within the CD3+ T cell population following administration of a single intravenous bolus dose of THOR-707 (0.3, 1, and 3 mg/kg) or Aldesleukin. (0.3 mg/kg). Blood was drawn via cardiac puncture at the indicated time points and immune cell populations were assessed by FACS. Each data point represents an average from replicates at each time point ± SEM. (n=3 animals) Source data are provided as a Source Data file.

Supplementary Figure 15. THOR-707 induced the expansion of peripheral blood NK cells yet minimally expanded Treg cells.

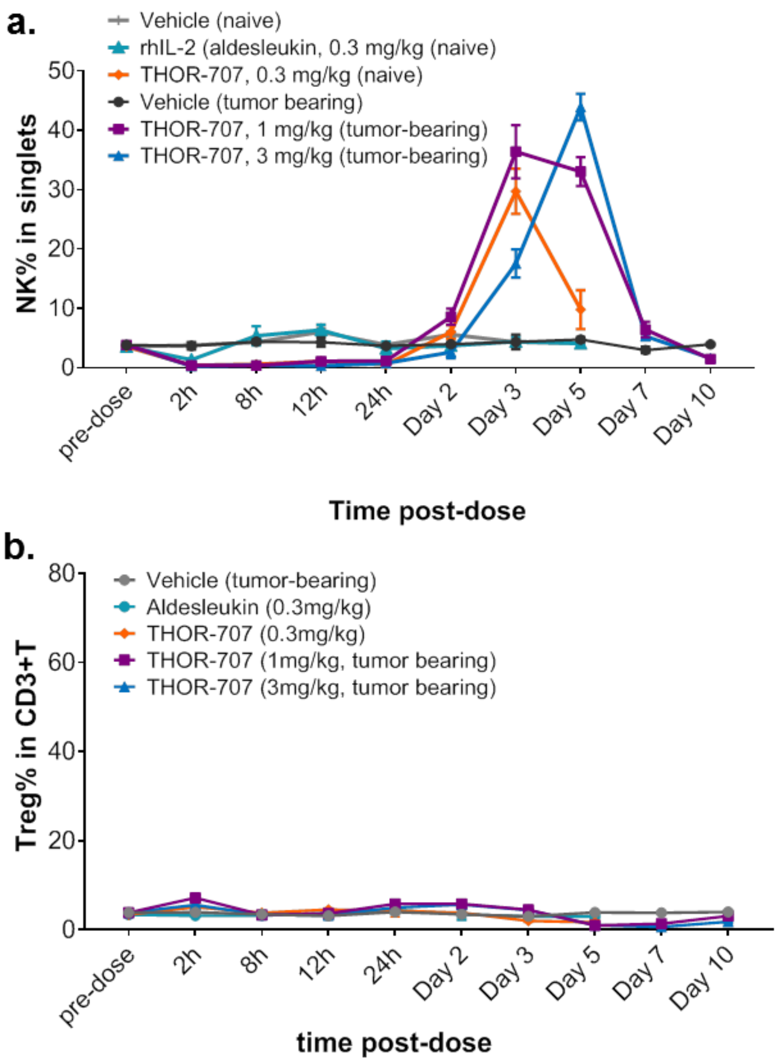

Supplementary Figure 15. THOR-707 induced the expansion of peripheral blood NK cells yet minimally expanded Treg cells. Percentage of peripheral blood (A) NK cells and (B) Treg cells following administration of a single intravenous bolus dose of THOR-707 (0.3, 1, or 3 mg/kg) or Aldesleukin. (0.3 mg/kg). Blood was drawn via cardiac puncture at the indicated time points and NK and Treg cell populations were assessed by FACS. Each data point represents an average from replicates at each time point ± SEM. (n=3 animals) Source data are provided as a Source Data file.

Supplementary Table 9. Analytical summary for custom nucleotides used in this study

Compound: dNaMTP (100mM)
Lot #: LN180808dNaM
Manufacturer: MyChem Inc (San Diego, CA)
Purity by HPLC 98.2%; 1H NMR (400 MHz, D2O): δ 8.08 (s, 1H), 7.96–7.97 (D, 1H), 7.88-7.89 (d, 1H), 7.53-7.56 (t, 1H), 7.45-7.48 (t, 1H), 7.41 (s, 1H), 5.60-5.63 (q, 1H), 4.78 (1H with H2O peak), 4.29 (m, 1H), 4.21 (m, 2H), 4.00 (s, 3H), 2.44-2.48 (m, 1H), 2.23-2.28 (m, 1H); 31P NMR: -5.21ppm (d, 1P), -9.92 ppm (d, 1P), -20.10 ppm (t, 1P); [M]+ calcd. for C16H21O13P3, 514.2; found, 513.1
Compound: NaMTP (100mM)
Lot #: LN181108NaM
Manufacturer: MyChem Inc (San Diego, CA)
Purity by HPLC 99.5%; 1H NMR (400 MHz, D2O): δ 8.10 (s, 1H), 7.97–7.97 (D, 1H), 7.85-7.87 (d, 1H), 7.53-7.55 (t, 1H), 7.43-7.47 (t, 1H), 7.39 (s, 1H), 5.31-5.32 (q, 1H), 4.78 (1H with H2O peak), 4.34-4.37 (m, 2H), 4.27-4.33 (m, 2H), 3.99 (s, 3H); 31P NMR: -4.79ppm (d, 1P), -10.20 ppm (d, 1P), -20.20 ppm (t, 1P); [M]+ calcd. for C16H21O15P3, 530.2; found,529.1
Compound: dTPT3TP (100mM)
Lot #: LN180402dTpT3
Manufacturer: MyChem Inc (San Diego, CA)
Purity by HPLC 98.0%; 1H NMR (400 MHz, D2O): δ 8.47-8.19 (d, 1H), 8.09–8.10 (D, 1H), 7.62-7.63 (d, 1H), 7.52-7.54 (d, 1H), 7.42-7.45 (t, 1H), 7.41 (s, 1H), 4.78 (1H with H2O peak), 4.35-4.38 (m, 2H), 2.77-2.82 (m, 1H), 2.36-2.42 (m, 1H); 31P NMR: -4.77ppm (d, 1P), -10.46 ppm (d, 1P), -20.27 ppm (t, 1P); [M]+ calcd. for C12H16NO12P3S2, 523.3; found, 522.1.
Compound: TPT3TP (100mM)
Lot #: LN180828TpT3
Manufacturer: MyChem Inc (San Diego, CA)
Purity by HPLC 98.5%; 1H NMR (400 MHz, D2O): δ 8.48-8.49 (d, 1H), 8.07–8.08 (D, 1H), 7.57-7. 58 (d, 1H), 7.49-7.50 (d, 1H), 7.13 (s, 1H), 4.78 (1H with H2O peak), 4.47-4.52 (m, 2H), 4.42-4.44 (m, 1H); 4.38-4.41 (m, 1H); 31P NMR: -5.73ppm (d, 1P), -10.52 ppm (d, 1P), -21.48 ppm (t, 1P); [M]+ calcd. for C12H16NO13P3S2, 539.3; found, 538.0.

Supplementary Table 10. Oligonucleotides used in this study

Primer Name	Sequence	Description
IL-2 K35 AXC oligo	GAACGGCATCAACAACTACAAAAATCCGA-NaM-CCTGACCCGTATGCTGACCTTCAAATTCTACATGCCGAAAAAAGCAACCGAGC	Unnatural oligonucleotide for generation of IL-2 K35 AXC Golden Gate Insert used in assembly of expression plasmid
IL-2 R38 AXC oligo	GAACGGCATCAACAACTACAAAAATCCGAAACTGACCA-NaM-CATGCTGACCTTCAAATTCTACATGCCGAAAAAAGCAACCGAGC	Unnatural oligonucleotide for generation of IL-2 R38 AXC Golden Gate Insert used in assembly of expression plasmid
IL-2 T41 AXC oligo	GAACGGCATCAACAACTACAAAAATCCGAAACTGACCCGTATGCTGA-NaM-CTTCAAATTCTACATGCCGAAAAAAGCAACCGAGC	Unnatural oligonucleotide for generation of IL-2 T41 AXC Golden Gate Insert used in assembly of expression plasmid
IL-2 F42 AXC oligo	GAACGGCATCAACAACTACAAAAATCCGAAACTGACCCGTATGCTGACCANCAAATTCTACATGCCGAAAAAAGCAACCGAGC	Unnatural oligonucleotide for generation of IL-2 F42 AXC Golden Gate Insert used in assembly of expression plasmid
IL-2 Y45 AXC oligo	GAACGGCATCAACAACTACAAAAATCCGAAACTGACCCGTATGCTGACCTTCAAATTCA-NaM-ATGCCGAAAAAAGCAACCGAGCTGAA	Unnatural oligonucleotide for generation of IL-2 Y45 AXC Golden Gate Insert used in assembly of expression plasmid
JP425 IL-2 K35-43 GG insert F	TATTGGTCTCTTCTGAACGGCATCAACAACTACAAAAATC	Forward primer for generation of IL-2 K35-43 Golden Gate Inserts used in assembly of expression plasmids
JP426 IL-2 K35-43 GG insert R	TATTGGTCTCTTTCAGCTCGGTTGCTTTTTTCGGCATG	Reverse primer for generation of IL-2 K35-43 Golden Gate Inserts used in assembly of expression plasmids
JP504 K35-43 GG insert R2	TATTGGTCTCTTTCAGCTCGGTTGCTTTTTTC	Reverse primer for generation of IL-2 K35-43 Golden Gate Inserts (sites T41, F42 and Y45) used in assembly of expression plasmids
JP427 IL-2 K35-43 GG vector F	GTACCGGTCTCCTGAAACATCTGCAGTGTCTG	Forward primer for generation of IL-2 K35-43 Golden Gate Entry Vector used in assembly of expression plasmids
JP428 IL-2 K35-43 GG vector R	CGGTCTCTCAGAATCATTTGCAGATC	Reverse primer for generation of IL-2 K35-43 Golden Gate Entry Vector used in assembly of expression plasmids
IL-2 E62 AXC oligo	GAGCTGAAACATCTGCAGTGTCTGGAAGAAA-NaM-CCTGAAACCGCTGGAAGAGGTTCTGAATCTGGCACAGAG	Unnatural oligonucleotide for generation of IL-2 E62 AXC Golden Gate Insert used in assembly of expression plasmid
THOR-707 IL-2 P65 AXC oligo	CATCTGCAGTGTCTGGAAGAAGAACTGAAAA-NaM-CCTGGAAGAGGTTCTGAATCTGGCACAGAGCAAAAACTTTCATCTGCG	Unnatural oligonucleotide for generation of THOR-707 IL-2 P65 AXC Golden Gate Insert used in assembly of expression plasmid
JP546 IL-2 E62-65 GG insert F	TATTGGTCTCAACCGAGCTGAAACATCTGCAGTGTC	Forward primer for generation of IL-2 E62-65 Golden Gate Inserts used in assembly of expression plasmids

JP547 IL-2 E62-65 GG insert R	TATTGGTCTCTTTGCTCTGTGCCAGATTCAGAACCTCTTCCCTGGAAGAGGTTCTGAATCTGGCACAGAGCAAAAACTTTCATCTGCG	Reverse primer for generation of IL-2 E62-65 Golden Gate Inserts used in assembly of expression plasmids
JP548 GG vector F	GTACCGGTCTCAGCAAAAACTTTCATCTGCGTCCGCGTG	Forward primer for generation of THOR-707 Golden gate entry vector used in assembly of expression plasmids
JP549 GG vector R	CGGTCTCTCGGTTGCTTTTTTCGGCATGTAG	Reverse primer for generation of THOR-707 Golden Gate entry vector used in assembly of expression plasmids
IL-2 E68 AXC oligo	GTCTGGAAGAAGAACTGAAACCGCTGGAAA-NaM-CGTTCTGAATCTGGCACAGAGCAAAAACTTTC	Unnatural oligonucleotide for generation of IL-2 E68 AXC Golden Gate Insert used in assembly of expression plasmid
IL-2 V69 AXC oligo	GTCTGGAAGAAGAACTGAAACCGCTGGAAGAGA-NaM-CCTGAATCTGGCACAGAGCAAAAACTTTC	Unnatural oligonucleotide for generation of IL-2 V69 AXC Golden Gate Insert used in assembly of expression plasmid
JP500 IL-2 E68-69 GG insert F	TATTGGTCTCTGTCTGGAAGAAGAACTGAAACCGCTG	Forward primer for generation of IL-2 E68-69 Golden Gate Inserts used in assembly of expression plasmids
JP501 IL-2 E68-69 GG insert R	TATTGGTCTCCGCAGATGAAAGTTTTTGCTCTGTGCCAGATTC	Reverse primer for generation of IL-2 E68-69 Golden Gate Inserts used in assembly of expression plasmids
JP502 IL-2 E68-69 GG vector R	CCGGTCTCCAGACACTGCAGATGTTTC	Reverse primer for generation of IL-2 E68-69 Golden Gate Inserts used in assembly of expression plasmids
JP503 IL-2 E68-69 GG vector F	TACCGGTCTCTCTGCGTCCGCGTGATCTGATTAG	Forward primer for generation of IL-2 E68-69 Golden Gate Inserts used in assembly of expression plasmids
Mm *pylT* U25C AXC GG template	GAATCTAACCCGGCTGAACGGATTA-NaM-CAGTCCGTTCGATCTACATGATCAGG	Unnatural oligonucleotide for generation of Mm *pylT* U25C AXC Golden Gate Insert used in assembly of expression plasmids
JP342 Mm *pylT* GG insert F	ATGGGTCTCGAAACCTGATCATGTAGATCGAACG	Forward primer for generation of Mm *pylT* Golden Gate Insert used in assembly of expression plasmids
JP343 Mm *pylT* GG insert R	ATGGGTCTCATCTAACCCGGCTGAAC	Reverse primer for generation of Mm *pylT* Golden Gate Insert used in assembly of expression plasmids

Supplementary Table 11. Antibodies used in flow cytometry assays for pSTAT5 induction in primary human PBMC

Antigen	Fluorophore	Clone	Vendor	Catalog #	dilution
CD4	BUV737	SK3	BD	564305	1:50
CD4	PE-Cy7	RPA-T4	Biolegend	300512	1:200
CD56	BV711	HCD56	Biolegend	318336	1:100
CD56	BV421	HCD56	Biolegend	318328	1:100
CD16	BV711	3G8	Biolegend	302044	1:100
CD8	BUV805	SK1	BD	564912	1:50
CD8	PerCP-Cy5.5	RPA-T8	BD	560662	1:100
CD27	BV786	L128	BD	563327	1:25
CD45RA	BUV395	HI100	BD	740298	1:50
CD45RA	A488	HI100	Biolegend	304114	1:500
CD127	FITC	eBioRDR5	eBioscience	11-1278-42	1:50
CD127	eF506	eBioRDR5	eBioscience	69-1278-42	1:50
CD25	Biotin	M-A251	Biolegend	356124	1:25
CD25	PE	M-A251	Biolegend	356104	1:500
CD3	PE-Cy7	UCHT1	Biolegend	300420	1:50
CD3	APC-Cy7	UCHT1	Biolegend	300426	1:500
STAT5	Ax647	47/Stat5(pY694)	BD	562076	1:20
Streptavidin	BV421	N/A	Biolegend	405225	1:200
FOXp3	PE	259D	Biolegend	320208	1:25

Supplementary Figure 16. Gating strategies for flow cytometry analysis of pSTAT5 induction in primary human PBMC.

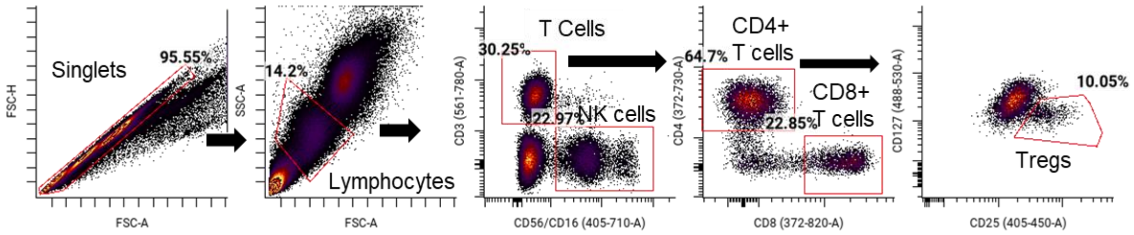

Gating strategies for flow cytometry analysis of pSTAT5 induction in primary human PBMC. The Flow Cytometry fcs files were gated on singlets using FSC-A by FSC-H to exclude any aggregates or doublets. Within this gate the cells were gated on mid to high forward scatter (FSC-A) and side scatter (SSC-A) to exclude the red blood cells, debris, and granulocytes (Lymphocyte gate). The T cells were then gated as the CD3+, CD56/16 negative population. The NK cells were identified as the CD3 negative, CD56/16 high population. The T cells were then divided into CD4+ T cells and CD8+ T cells. Finally, Tregs were gated from the CD4+ T cells as the CD25hi x C127lo population.

Supplementary Table 12. Antibody panel used to profile the immune cell phenotyping from mouse whole blood samples.

Antigen	Fluorophore	Clone	Vendor	Catalog #	dilution
CD3	Ax488	17A2	Biolegend	100210	1:400
CD4	Bv786	RM4-5	Biolegend	100552	1:200
CD8	Bv711	53-6.7	Biolegend	100759	1:100
NK1.1	Bv421	PK136	Biolegend	108741	1:25
CD25	Biotin	REA568	Miltenyi	130-108-995	1:25
FoxP3	PE	FJK-16s	Invitrogen	12-5773-82	1:25
CD44	PEcy7	IM7	Biolegend	103030	1:1000
Ki67	PerCP eFluor 710	SolA15	Invitrogen	46-5698-82	1:500
Streptavidin	BUV395	-	BD	564176	1:1000
pStat5	Ax647	47/Stat5 (pY694)	BD	612599	1:5

Supplementary Table 13. Antibody panel used to profile the immune cell phenotyping for tumor and spleen samples in B16-F10 tumor PK/PD study.

Antigen	Fluorophore	Clone	Vendor	Catalog#	µg/mL
CD45	PEcy7	30-F11	Biolegend	103114	2.5
CD3e	BUV395	17A2	BD	740268	2
CD4	APC-eF780	GK1.5	eBioscience	47-0041-80	1.25
CD8a	PE-eF610	53-6.7	eBioscience	61-0081-82	5
CD335	BV605	29A1.4	Biolegend	137619	7
CD44	BV421	IM7	Biolegend	103040	2.5
Foxp3	PE	FJK-16s	eBioscience	12-5773-82	10
L/D	eFluor 506	-	eBioscience	65-0866-18	-
CD122	BV711	TM-Beta 1	BD	740679	2
CD62L	Percpcy5.5	MEL-14	Biolegend	104412	2.5
Ki67	AF488	11F6	Biolegend	151204	2.5
CD49b	APC	DX5	Biolegend	108910	2.5
CD25	AF700	PC61	Biolegend	102024	2.5

The combination of the following biomarkers were used to identify the cell population reported in this study.

Cell Type	Marker Profile
Effector T cells (Teff)	CD3+, CD4+, CD8+
CD8+ memory cells	CD3+, CD8+, CD44+
NK cells	CD3-, CD335+ CD49b+
Regulatory T cells (Treg)	CD3+, CD4+, CD8-, CD25+, FoxP3+
Proliferation	Ki67+
