# Supplementary material for: An engineered IL-2 reprogrammed for anti-tumor therapy using a semi-synthetic organism
Source: Nat Commun. 2021 Aug 9;12:4785. doi: 10.1038/s41467-021-24987-9 (PMC8352909; doi:10.1038/s41467-021-24987-9)
Supplement: Supplementary file 2 — Reporting Summary [file 41467_2021_24987_MOESM2_ESM.pdf]

nature research

Corresponding author(s): Jerod Ptacin and Marcos Milla

Last updated by author(s): June 28, 2021

Reporting Summary

Nature Research wishes to improve the reproducibility of the work that we publish. This form provides structure for consistency and transparency in reporting. For further information on Nature Research policies, see our Editorial Policies and the Editorial Policy Checklist.

Statistics

For all statistical analyses, confirm that the following items are present in the figure legend, table legend, main text, or Methods section.

n/a	Confirmed	
☐	☒	The exact sample size (*n*) for each experimental group/condition, given as a discrete number and unit of measurement
☐	☒	A statement on whether measurements were taken from distinct samples or whether the same sample was measured repeatedly
☐	☒	The statistical test(s) used AND whether they are one- or two-sided<br>*Only common tests should be described solely by name; describe more complex techniques in the Methods section.*
☒	☐	A description of all covariates tested
☒	☐	A description of any assumptions or corrections, such as tests of normality and adjustment for multiple comparisons
☐	☒	A full description of the statistical parameters including central tendency (e.g. means) or other basic estimates (e.g. regression coefficient) AND variation (e.g. standard deviation) or associated estimates of uncertainty (e.g. confidence intervals)
☐	☒	For null hypothesis testing, the test statistic (e.g. *F*, *t*, *r*) with confidence intervals, effect sizes, degrees of freedom and *P* value noted<br>*Give P values as exact values whenever suitable.*
☒	☐	For Bayesian analysis, information on the choice of priors and Markov chain Monte Carlo settings
☒	☐	For hierarchical and complex designs, identification of the appropriate level for tests and full reporting of outcomes
☒	☐	Estimates of effect sizes (e.g. Cohen's *d*, Pearson's *r*), indicating how they were calculated

Our web collection on statistics for biologists contains articles on many of the points above.

Software and code

Policy information about availability of computer code

Data collection	Flow cytometry- FlowJo v10<br>Plate reader (Discoverx study)- PerkinElmer EnvisionTM<br>Biacore (SPR) software package<br>Plate reader (PK analysis)- SoftMax Pro (v7.1)
Data analysis	Structure analysis and alignment- Pymol v2.3.0<br>PK analysis- Phoenix WinNonlin 8.1<br>SPR analysis- Scrubber v2.0c<br>Flow cytometry analysis, fitting- Spotfire v7.14<br>Flow cytometry analysis, CellEngine (PrimityBio)<br>Plotting and statistical analysis- GraphPad Prism 8 XML<br>Crystallography- REFMAC (5.8.0155)<br>Crystallography- CCP4 7.0.020<br>Crystallography- Coot 0.9.5<br>Crystallography- autoPROC, XDS (VERSION Jan 26, 2018)<br>Crystallography- autoPROC (Version 1.1.7), AIMLESS (version 0.5.27)<br>Discoverx IL-2 potency data fit statistics- CBIS data analysis suite (ChemInnovation, CA)

For manuscripts utilizing custom algorithms or software that are central to the research but not yet described in published literature, software must be made available to editors and reviewers. We strongly encourage code deposition in a community repository (e.g. GitHub). See the Nature Research guidelines for submitting code & software for further information.

Data

Policy information about availability of data

All manuscripts must include a data availability statement. This statement should provide the following information, where applicable:

- Accession codes, unique identifiers, or web links for publicly available datasets
- A list of figures that have associated raw data
- A description of any restrictions on data availability

Source data are provided with this paper. The datasets generated and/or analysed during the studies reported herein are provided in the Supplementary Information and in the Source Data file, provided for Figures 1, 3, 4, 5, 6, Supplementary Figures 1, 5-14. The structure factors and coordinates for the rhIL-2 (P65K) structure have been deposited into the Protein Data Bank under the accession number 7M2G (https://doi.org/10.2210/pdb7M2G/pdb). The coordinates for rhIL-2 bound to the heterotrimeric IL-2 receptor complex structure used for structural alignments is publicly available (2ERJ, DOI: 10.2210/pdb2ERJ/ pdb).

Field-specific reporting

Please select the one below that is the best fit for your research. If you are not sure, read the appropriate sections before making your selection.

☒ Life sciences ☐ Behavioural & social sciences ☐ Ecological, evolutionary & environmental sciences

For a reference copy of the document with all sections, see nature.com/documents/nr-reporting-summary-flat.pdf

Life sciences study design

All studies must disclose on these points even when the disclosure is negative.

Sample size

Sample sizes were not statistically pre-determined.
Appropriate sample sizes were selected for each experiment to allow meaningful statistical confidence where required
For initial screening studies using the Discoverx assay, each compound was tested in duplicate to ensure reproducibility to identify potential hit compounds and discern from pharmacologically inappropriate variants. Duplicate measurements were considered sufficient for screening as the density of sampling across doses ensured reasonable accuracy of curve fits sufficient to identify large differences in potency. For pSTAT5 flow studies, sample size were selected to minimize natural donor variability effects and allow mean and SEM calculations for potency data. For in vivo studies, sample size was selected to balance animal use with statistical power, and allow temporal sampling density required to observed the effects in cases where terminal collections were required (PK/PD studies). For PK/PD studies, at least three animals per time point per compound were selected as appropriate to minimize animal to animal variability and statistical measure of the standard error of the mean effect. For tumor studies, sample sizes were selected to balance animal use and statistical power, and to minimize animal to animal variability effects on the results.

Data exclusions

In the SPR binding study, rhIL-2 concentrations of 5-10uM and above produced aberrant signals that were not able to be fit. These signals saturated the surface and were excluded from the analysis to avoid artifacts in the fitting.

For other studies, data were excluded when a sample failed due to a technical issue, and exclusion criteria were established before data were collected. All such exclusions are listed in the Source Data file and summarized below.

Fig 5c, one data point was omitted from each of THOR-707 1mg/kg and 3mg/kg samples (n=3 for these vs n=4 for all others).*
Supplementary Figure 7. Two data points were omitted from the THOR-707 1mg/kg B16-F10 Percent pSTAT5 positive in NK cells analysis.*
Supplementary Figure 8. Two data points were omitted from different time points from each of vehicle, THOR-707 and rhIL-2 data sets, as well as the 120 hours data from all sets.*
Supplementary Figure 9 B. Percent Ki-67 positive in NK cells from B16F10 tumor-bearing and non-tumor bearing C57BL6 mice. Two data points were omitted from the 3mg/kg data set and one from the 0.3mg/kg data set.*
Supplementary Figure 14. One data point was omitted from each of the following groups: THOR-707 (0.3mg/kg) in C57BL6 (non tumor bearing mice), THOR-707 1mg/kg in C57BL6 mice bearing B16F10 tumors , THOR-707 3mg/kg in C57BL6 mice bearing B16F10 tumors.

* exclusion based on low event count in flow cytometry analysis (<100 events in target cell population). Criteria for event counts were >100 events in target population. All such exclusions are highlighted in the Source Data file

Replication

All data produced was based on biologically independent samples with the exception of the Discoverx screen, in which technical replicates were employed to ensure technical reproducibility for screening. No technical replicates were used for statistical measurements.
In vitro studies were performed with multiple donors where applicable and described in methods section.
All attempts at replication were successful.

Discoverx screening of IL-2 variants was performed in duplicate. Results for THOR-707 were confirmed in >2 additional independent studies.
THOR-707 vs IL-2 SPR was performed in singlicate. Absence of IL-2Ra engagement was confirmed in >2 additional independent studies.
Ex vivo potency via Flow cytometry was performed in singlicate using six independent donors. Results for THOR-707 were confirmed in >2 additional studies with different random donors.
Single dose PK/PD study in naive mice was performed in singlicate using 4 animals per time point. THOR-707 CD8+T and NK expansion, and

absence of Treg expansion were confirmed in >2 additional studies.
PK/PD study in tumor bearing mice was performed in singlicate using 4-7 animals per time point.
B16-F10 tumor efficacy study was performed in singlicate using 15 animals per condition.
PBMC dose response for pSTAT5 study (Supplementary Fig 5) was performed in singlicate with 3 independent donor samples.
Supplementary Fig 11- IL-5 levels after rhIL-2 or THOR-707 administration was performed in singlicate in 3 independent animals per time point

Randomization

Samples/animals in all experiments were randomized to each group.
In PK/PD study in naive mice, animals were assigned to each group at random.
In B16F10 tumor PK/PD study, mice were randomly assigned to groups for treatment. Randomization was performed based on average tumor volume (mm3) at ~60-80mm3 for each group on day -1 (a day prior to dosing).
In B16F10 efficacy study, mice were randomized at tumor volumes of 50 mm3 for the treatment. All animals were randomly allocated to the different study groups.
For PK/PD studies in mouse, mice were allocated to study groups at random.

Blinding

All studies reported herein report quantitative data measured without subjective scoring. The investigators were not blinded during experiments

Reporting for specific materials, systems and methods

We require information from authors about some types of materials, experimental systems and methods used in many studies. Here, indicate whether each material, system or method listed is relevant to your study. If you are not sure if a list item applies to your research, read the appropriate section before selecting a response.

Materials & experimental systems

n/a	Involved in the study
☐	☒ Antibodies
☐	☒ Eukaryotic cell lines
☒	☐ Palaeontology and archaeology
☐	☒ Animals and other organisms
☐	☒ Human research participants
☒	☐ Clinical data
☒	☐ Dual use research of concern

Methods

n/a	Involved in the study
☒	☐ ChIP-seq
☐	☒ Flow cytometry
☒	☐ MRI-based neuroimaging

Antibodies

Antibodies used

All antibodies used in the studies reported are described in detail in the supplemental materials section of the manuscript and are listed below.

CD122 BV711 TM-Beta 1 BD 740679
CD127 FITC eBioRDR5 eBioscience 11-1278-42 1:50
CD127 eF506 eBioRDR5 eBioscience 69-1278-42 1:50
CD16 BV711 3G8 Biolegend 302044 1:100
CD25 Biotin REA568 Miltenyi 130-108-995 1:25
CD25 AF700 PC61 Biolegend 102024
CD25 Biotin M-A251 Biolegend 356124 1:25
CD25 PE M-A251 Biolegend 356104 1:500
CD27 BV786 L128 BD 563327 1:25
CD3 Ax488 17A2 Biolegend 100210 1:400
CD3 PE-Cy7 UCHT1 Biolegend 300420 1:50
CD3 APC-Cy7 UCHT1 Biolegend 300426 1:500
CD335 BV605 29A1.4 Biolegend 137619
CD3e BUV395 17A2 BD 740268
CD4 Bv786 RM4-5 Biolegend 100552 1:200
CD4 APC-eF780 GK1.5 eBioscience 47-0041-80
CD4 BUV737 SK3 BD 564305 1:50
CD4 PE-Cy7 RPA-T4 Biolegend 300512 1:200
CD44 PEcy7 IM7 Biolegend 103030 1:1000
CD44 BV421 IM7 Biolegend 103040
CD45 PEcy7 30-F11 Biolegend 103114
CD45RA BUV395 HI100 BD 740298 1:50
CD45RA A488 HI100 Biolegend 304114 1:500

CD49b APC DX5 Biolegend 108910
CD56 BV711 HCD56 Biolegend 318336 1:100
CD56 BV421 HCD56 Biolegend 318328 1:100
CD62L Percpcy5.5 MEL-14 Biolegend 104412
CD8 Bv711 53-6.7 Biolegend 100759 1:100
CD8 BUV805 SK1 BD 564912 1:50
CD8 PerCP-Cy5.5 RPA-T8 BD 560662 1:100
CD8a PE-eF610 53-6.7 eBioscience 61-0081-82
FoxP3 PE FJK-16s Invitrogen 12-5773-82 1:25
FOXp3 PE 259D Biolegend 320208 1:25
Ki67 PerCP eFluor 710 SolA15 Invitrogen 46-5698-82 1:500
Ki67 AF488 11F6 Biolegend 151204
L/D eFluor 506 - eBioscience 65-0866-18
NK1.1 Bv421 PK136 Biolegend 108741 1:25
pStat5 Ax647 47/Stat5 (pY694) BD 612599 1:5 or 1:20
Streptavidin BUV395 BD 564176 1:1000
Streptavidin BV421 Biolegend 405225 1:200
Anti-IL-2 oligoclonal antibody, rabbit anti-human Novex 710146 1:2500 (Western Blot)

Validation

All antibodies used for the studies reported are commercially available, each lot was quality control tested and has been validated by the manufacturer for the intended use. For immunophenotyping of immune cells, commonly used markers for individual populations were chosen (ie. CD3 for T cells etc). Antibodies were titrated for optimal performance where necessary. For Western Blotting, antibodies were validated for sensitivity and specificity using rhIL-2 standards and negative control samples.

Eukaryotic cell lines

Policy information about cell lines

Cell line source(s)

U2OS (human)- platform/parental cell line for Discoverx PathHunter assay
B16F10 (murein)- American Tissue Type Collection (ATCC, Manassas, VA)- Crown Bio

Authentication

Cell lines obtained from reputable suppliers and not independently authenticated to our knowledge

Mycoplasma contamination

Cell lines obtained from reputable suppliers and not independently tested to our knowledge

Commonly misidentified lines (See ICLAC register)

No commonly misidentified cell lines were used in the study.

Animals and other organisms

Policy information about studies involving animals; ARRIVE guidelines recommended for reporting animal research

Laboratory animals

C57BL/6 female mice 6-8 weeks of age with an average weight of 16 to 22 grams were purchased from Jackson Laboratories (Sacramento, CA) by Crown Biosciences. As stated in the manuscript, C57BL6 mice were housed at 72.5 Deg F, ambient humidity, and 12 hours light : dark cycle.

Wild animals

No wild animals were used in the study.

Field-collected samples

No field collected samples were used in the study.

Ethics oversight

In vivo studies were conducted by qualified personnel by Crown Biosciences. Animal welfare for these studies complies with the U.S. Department of Agriculture's Animal Welfare Act (9 CFR Parts 1, 2 and 3) as applicable. All experimental data management and reporting procedures were in strict accordance with applicable Crown Bioscience, Inc. Guidelines and Standard Operating Procedures.

Note that full information on the approval of the study protocol must also be provided in the manuscript.

Human research participants

Policy information about studies involving human research participants

Population characteristics

Human blood was collected from anonymous healthy donor volunteers.

Recruitment

Human blood samples we purchased from iXcells, Stanford University, or obtained from the Scripps Research institute.

Ethics oversight

Primary human PBMC potency studies using flow cytometry were performed under contract by PrimityBio (Fremont CA) or internally. PrimityBio purchased human blood samples through an IRB with Stanford University (Reg#: 5136 (eprotocol 13942) and 6208 (eprotocol 38735)). Internal blood samples collection from anonymous healthy donors were approved by

the Institutional Review Board of The Scripps Research Institute (#177065). Leukocyte reduction systems (LRS) were purchased from Cell IDX (San Diego, CA). All subjects provided informed consent. The studies were performed following the guidelines of the World Medical Association's Declaration of Helsinki.

Note that full information on the approval of the study protocol must also be provided in the manuscript.

Flow Cytometry

Plots

Confirm that:

- [x] The axis labels state the marker and fluorochrome used (e.g. CD4-FITC).
- [x] The axis scales are clearly visible. Include numbers along axes only for bottom left plot of group (a 'group' is an analysis of identical markers).
- [x] All plots are contour plots with outliers or pseudocolor plots.
- [x] A numerical value for number of cells or percentage (with statistics) is provided.

Methodology

Sample preparation	Whole blood samples were treated with 20 volumes of pre-warmed Lyse/Fix Buffer (BD Phosflow™, catalog #558049) and incubated for 10 min at 37°C.<br>Tumor samples for flow cytometry were processed immediately after collection. MACS mouse tumor dissociation kit (Miltenyi Biotec) was used to process tumor samples into single cells for flow cytometry analysis.
Instrument	Becton Dickinson Fortessa, LSRII, or ThermoFisher Attune NxT instruments
Software	FCS files were analyzed using CellEngine, a browser-based flow cytometry analysis program by PrimityBio, or FlowJo v 10.
Cell population abundance	Data available upon request
Gating strategy	FCS files are gated on singlets using FSC-A by FSC-H to exclude any aggregates or doublets. Within this gate the cells are gated on mid to high forward scatter (FSC-A) and side scatter (SSC-A) to exclude the red blood cells, debris, and granulocytes. In some studies, live/dead viability stain is used to exclude dead cells and CD45 is used to separate leukocytes from red blood cells and debris. T cells are then gated as the CD3+, CD56/16 negative population. NK cells are identified as the CD3 negative, CD56/16 high population. In mouse studies, NK cells are defined as NK1.1 positive. T cells are then divided into CD4+ T cells and CD8+ T cells. Tregs are then gated from the CD4+ T cells as the CD25hiC127lo or CD25+FoxP3+ population.

- [x] Tick this box to confirm that a figure exemplifying the gating strategy is provided in the Supplementary Information.
